# Down-regulation of *coasy*, the gene associated with NBIA-VI, reduces Bmp signaling, perturbs dorso-ventral patterning and alters neuronal development in zebrafish

**DOI:** 10.1038/srep37660

**Published:** 2016-11-28

**Authors:** Deepak Khatri, Daniela Zizioli, Natascia Tiso, Nicola Facchinello, Sara Vezzoli, Alessandra Gianoncelli, Maurizio Memo, Eugenio Monti, Giuseppe Borsani, Dario Finazzi

**Affiliations:** 1 Department of Molecular and Translational Medicine, University of Brescia, viale Europa 11, 25123 Brescia, Italy; 2 Department of Biology, University of Padova, via U. Bassi 58/B, 35131, Padova, Italy; 3 Clinical Chemistry Laboratory, ASST Spedali Civili di Brescia, 25123 Brescia, Italy

## Abstract

Mutations in Pantothenate kinase 2 and Coenzyme A (CoA) synthase (*COASY*), genes involved in CoA biosynthesis, are associated with rare neurodegenerative disorders with brain iron accumulation. We showed that zebrafish *pank2* gene plays an essential role in brain and vasculature development. Now we extended our study to *coasy*. The gene has high level of sequence identity with the human ortholog and is ubiquitously expressed from the earliest stages of development. The abrogation of its expression led to strong reduction of CoA content, high lethality and a phenotype resembling to that of dorsalized mutants. Lower doses of morpholino resulted in a milder phenotype, with evident perturbation in neurogenesis and formation of vascular arborization; the dorso-ventral patterning was severely affected, the expression of bone morphogenetic protein (Bmp) receptors and activity were decreased, while cell death increased. These features specifically correlated with the block in CoA biosynthesis and were rescued by the addition of CoA to fish water and the overexpression of the human wild-type, but not mutant gene. These results confirm the absolute requirement for adequate levels of CoA for proper neural and vascular development in zebrafish and point to the Bmp pathway as a possible molecular connection underlining the observed phenotype.

Coenzyme A (CoA) is an essential cofactor in all living organisms being involved in about 4% of cellular biochemical processes. Its level is regulated by different extracellular stimuli and affected by various pathological conditions^1^,^2^. The biochemical steps of cellular CoA biosynthesis include five reactions. Pantothenic acid is firstly phosphorylated by pantothenate kinase (PANK) to generate 4′-phosphopantothenic acid (4′-PPA). Then, 4′-phosphopantothenoylcysteine synthase condenses cysteine with 4′-PPA forming 4′-phosphopantothenoylcysteine, which is subsequently decarboxylated to 4′-phosphopantetheine (4′-PP) by phosphopantothenoylcysteine decarboxylase. Finally, a bifunctional enzyme, CoA synthase (COASY), catalyzes the last two steps leading to the conversion of 4′-PP to dephospho-CoA and subsequently CoA^3^. The reactions controlled by PANK and CoA synthase are the limiting steps in the regulation of the whole process^4^. Quite unexpectedly, this fundamental biochemical pathway has been recently linked to cases of early onset neurodegeneration belonging to the Neurodegeneration with Brain Iron Accumulation (NBIA) category. This is a heterogeneous group of genetic neurologic disorders, characterized by dystonia, parkinsonism and spasticity, often with early onset and accumulation of iron in the brain, more typically in the basal ganglia^5^. In 2001, Zhou and colleagues^6^ identified nucleotide variations in the gene coding for PANK2 protein, one of the human isoforms of such enzyme. Pantothenate Kinase Associated Neurodegeneration (PKAN, MIM 234200) is the most common form of NBIA, representing almost two thirds of the cases included in this heterogeneous category^7^. More recently, mutations in *COASY* were found in patients with clinical manifestation and magnetic resonance imaging (MRI) signs typical of NBIA (MIM 615643)^8^,^9^. One subject carried a homozygous nucleotide substitution, c.1495 C > T, that affected a conserved arginine (p.Arg499Cys) in the nucleotide binding site of the dephospho-CoA kinase (DPCK) domain. The other case was a compound heterozygous carrying the same c.1495 C > T transition and the c.175 C > T variation, leading to a premature p.Gln59* stop codon in the N-terminal regulatory region of the protein. In both cases the mutations were associated with significant reduction of protein levels and enzymatic activity. Quite surprisingly, acetyl-CoA but not total CoA levels were significantly different in fibroblasts from patients versus controls and *de-novo* CoA biosynthesis was clearly reduced, but not absent.

The association between defects in PANK2 and COASY and specific types of NBIA obviously suggests a central role for CoA metabolism in neural cell development and maintenance, although the pathogenic mechanisms underlining this connection are not defined yet. The simplest interpretation of existing evidences links defects in PANK2 and COASY with shortage of cellular CoA that, in turn, acts as initial trigger of a cascade of events culminating with neural death in specific brain areas. This hypothesis is supported by the rescue capacity of pantethine and CoA documented both in cellular and animal models^10^,^11^,^12^,^13^. On the other hand, measurement of CoA concentrations in fibroblasts or blood samples from patients did not evidence reduced levels of the metabolite^8^,^14^. While this result is hard to explain for COASY mutants, compensating mechanisms can exist for PANK2 defects, since mammalian cells express other isoforms of the enzyme (PANK1a and b and PANK3). It may well be that distinctive features of PANK2, and particularly its localization in the mitochondrial intermembrane space, play a relevant role in determining the starting pathogenic mechanism and the specificity of PKAN neuropathology. Interestingly, morphological and functional perturbations of mitochondria have been found in fibroblasts from patients as well as in *Drosophila* and mouse knock-out models^10^,^15^,^16^. These features are often associated with signs of lipid dyshomeostasis, implying possible perturbation in mitochondrial membrane remodeling, and disruption of iron balance^15^,^17^. To gain new information about the functional connection between enzyme defects and pathology, we recently performed a thorough analysis of *pank2* role during zebrafish embryonic development^13^. When we down-regulated larval *pank2* expression by microinjection of a specific splice-inhibiting morpholino oligomer we observed a drastic reduction in *neurogenin1 neurog1* and *neurod1* positive neurons, particularly in telencephalon and midbrain, impaired development of main arteria, vein and intersegmental vessels and edema both in brain and at the tail plexus. Since defects of PANK2 and COASY affect the same biochemical pathway, we reasoned that a comparison of their involvement during zebrafish development could provide functional details about their specific role in the disease pathogenesis. To investigate the role of *coasy* during zebrafish development we microinjected a specific splice-inhibiting morpholino at 2-cell stage and followed the morphological consequences up to 72 hpf. At high morpholino doses the expression of *coasy* appeared completely abrogated and this resulted in a drastic decrease of CoA content and perturbation of embryos morphology, with severe reduction of the antero-posterior axis. At lower morpholino doses, the expression of *coasy* was reduced to about 60% and a milder phenotype was evident, partially resembling the one observed in *pank2* morphants. The use of a transgenic bone morphogenetic protein (Bmp)-reporter line evidenced a relevant reduction of *bmp* activation, associated with lower levels of different Bmp-receptor isoforms, a severe perturbation in antero-posterior patterning, somitogenesis, and neurogenesis, as evidenced by *in situ* hybridization with a variety of markers. This complex phenotype was rescued by treatment of embryos with CoA in fish water or by injection of the human *COASY* mRNA. The study demonstrates the essential role of *coasy* and CoA biosynthesis in normal zebrafish development but also indicates that neurogenesis and Bmp signaling are particularly sensitive to partial CoA depletion. This piece of information could be of relevance for the understanding of the specific phenotype and pathogenic process associated with mutations of the human *COASY* gene.

## Results

### Identification and *in silico* analysis of the *coasy* gene in zebrafish

The Gene and HomoloGene databases at NCBI^18^ indicate the presence of one putative ortholog of the human *COASY* gene in zebrafish, namely *coasy*, on the reverse strand of chromosome 24. This finding was confirmed by a homology-based search performed with the human COASY protein sequence (NP_001035997.2, 593 aa, c isoform) versus the latest assembly of zebrafish genomic sequences (Genome Reference Consortium Zebrafish Build 10, Sept. 2014).

The *Danio rerio coasy* gene (ZFIN Id: ZDB-GENE-040912-137, Ensembl Id: ENSDARG00000042747), similarly to the human one, is organized in ten coding exons. It expresses a main transcript isoform encoding for a 554 aa protein (NP_001004577.1) with 51% identity and 69% sequence similarity to the human counterpart (Supplementary Fig. S1). The analysis of the chromosomal regions surrounding the human and zebrafish genes, carried out using the Synteny Database^19^, showed a conserved synteny between the human chromosome 17 region harboring the *COASY* gene and *Danio rerio* chromosome 24 region where the ortholog is located (Supplementary Fig. S1). A single *coasy* gene is present in other teleosts (data not shown). Analysis of the UniGene database allowed the retrieval of 25 Expressed Sequence Tag (EST) derived from *coasy* zebrafish transcripts, indicating that the gene is expressed in different tissues and developmental stages. RNA-Seq data from the Wellcome Trust Sanger Institute Zebrafish Transcriptome Sequencing Project^20^ evidence the presence of RNA-Seq reads at all developmental stages analyzed, including 2-cell stage embryos, indicating that the gene is maternally expressed (data not shown).

### Temporal and spatial expression pattern of *coasy* during zebrafish development

We investigated the spatial and tissue-specific localization of *coasy* by whole mount mRNA *in situ* hybridization (WISH) in embryos from 0.2 to 36 hpf (Supplementary Fig. S2). A probe signal was detected since the earliest stages of development. At the sphere stage, *coasy* was expressed broadly in the entire blastoderm and, at the end of gastrulation, in ventral marginal and animal regions (presumptive ventral mesoderm and non-neural ectoderm, respectively) and, finally, in the animal pole and dorsal region. At the 20-somite stage, *coasy* displayed strong expression throughout the whole body but particularly in developing central nervous system (CNS) and somites. At later stages (24 and 36 hpf) the probe signal was detectable in specific brain regions, such as hindbrain, midbrain-hindbrain boundary, hindbrain ventricle tegmentum and persisted in newly formed somites. A sense probe was used in parallel control experiments at all stages without detecting any staining (Supplementary Fig. S2).

The expression of *coasy* gene was also investigated by real time RT-PCR. C*oasy* mRNA was detectable as early as 2-cell stage. Subsequently, its level remained rather constant throughout development with a slight decrease from 24 to 72 hpf (Supplementary Fig. S3). We then evaluated *coasy* expression in organs dissected from adult zebrafish, detecting ubiquitous expression, with higher levels in liver, muscles, eyes and brain (Supplementary Fig. S3). These results are in good agreement with available RNA Seq data. Overall, our study reveals that zebrafish *coasy* is a maternal gene being detected since the earliest stages of development. Consistent with the central role in cellular metabolism, its expression is rather ubiquitous throughout the stages of development and in adult organs, and more abundant in liver, muscles and head.

### Knock-down of *coasy* expression in zebrafish

**Figure 1:**
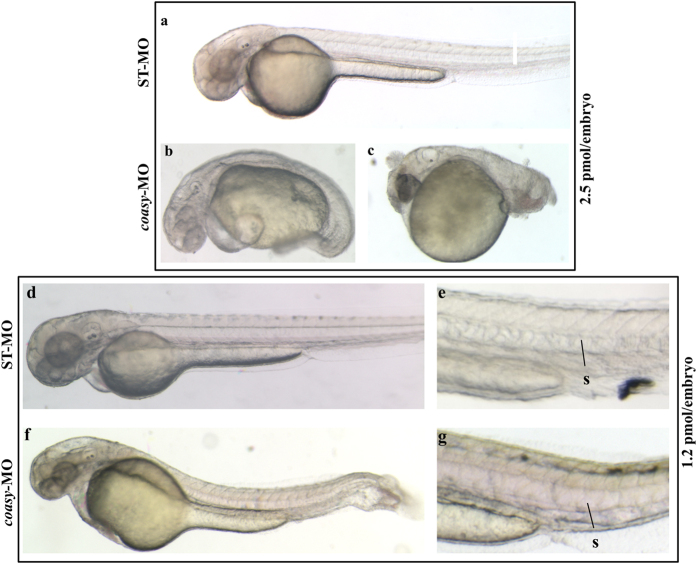
Phenotypic changes due to *coasy*-MO microinjection. Representative images of embryos injected with 2.5 or 1.2 pmol/embryo of ST-MO (**a,d,e**) or *coasy*-MO (**b,c,f,g**) at 48 hpf. Embryos injected with high doses of *coasy*-MO showed a severely aberrant phenotype [(B, 64%) and (C, 36%)]. Embryos injected with 1.2 pmol/embryo of *coasy*-MO (**f,g**) showed a milder phenotype.

**Figure 2:**
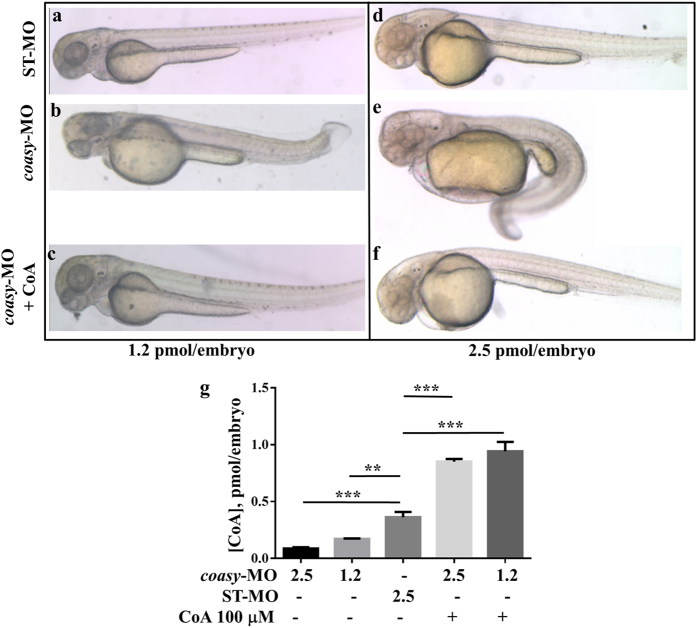
Morphological rescue by treatment with CoA. Morphological comparison of ST-MO- (**a,d**), *coasy*-MO-injected embryos (**b,e**) and morphants treated with 100 μM CoA at 48 hpf (**c,f**). Embryos were injected with 1.2 (**a–c**) or 2.5 pmol/embryo (**d–f**). Embryos exposed to CoA showed a significant correction of the aberrant phenotype, in particular the antero-posterior development, brain and somites formation. All images are lateral views. The graph in panel G shows the mean CoA content (pmol/embryo) as measured by a specific LC-MS/MS method on extracts from embryos injected with ST-MO or *coasy*-MO, eventually treated with 100 μM CoA.

We designed an antisense morpholino (*coasy*-MO) complementary to intron 1-exon 2 junction in the *coasy* gene to specifically inhibit the correct mRNA splicing and hence reduce Coasy protein level since the zygotic stage, without affecting the very early maternal expression. In parallel, we used a standard morpholino (ST-MO, Gene Tools, USA) with no target sequence in the zebrafish genome as negative control. In a dose-curve experiment (0.5 to 2.5 pmol/embryo), we observed a survival rate of about 70% in embryos injected with *coasy*-MO at 1.0, 1.2, 1.5 pmol/embryo. Higher doses (2.0–2.5 pmol/embryo) resulted in a significant mortality (more than 50%) (Supplementary Fig. S4). The effects of morpholino injection upon *in vivo* mRNA processing were verified by analyzing the specific mature mRNA by RT-PCR at 24 hpf. Primers were designed to amplify an 852 bp cDNA fragment from normally-spliced *coasy* mRNA and a shorter one (281 bp) when exon 2 was skipped. In embryos injected with higher doses of *coasy*-MO the longer band was absent and completely substituted by the shorter one, thus indicating a complete abrogation of *coasy* expression. At lower doses (from 0.5 to 1.5 pmol/embryo) both bands were visible with different intensities, indicating an incomplete suppression of the normal splicing (Supplementary Fig. S4). The doses of 2.5 and 1.2 pmol/embryo were selected to investigate the morphological changes induced by total or partial knock-down of *coasy* expression at 24 and 48 hpf. At 2.5 pmol/embryo only about 40% of embryos survived till 48 hpf; the vast majority of surviving embryos (79%, n = 211/263 from at least three independent experiments) exhibited a severely aberrant phenotype with marked alteration of the antero-posterior axis and features recalling either a class 3 (64%, n = 134/211) (Fig. 1b) or a class 4 (36%, n = 77/211) (Fig. 1c) dorsalized mutant^21^,^22^. In general, structures of ventral origin in the blastoderm were altered or absent whereas the anteriormost head regions were better conserved albeit not normal. The trunk was twisted or reduced with abnormal somites, the tail and posterior somites were absent and the yolk significantly enlarged in most embryos; at the same time also the head development was perturbed, with poor definition of most brain structures. We developed a specific Liquid Chromatography/Mass Spectrometry (LC-MS/MS) method to measure CoA content in embryos (Supplementary Fig. S5). As expected, the absence of *coasy* led to a significant reduction of CoA levels (0.087 vs 0.36 pmol/embryo, P < 0.001, Fig. 2g) and appeared to be not compatible with normal embryonic development and survival into adulthood. Interestingly, the observed phenotype let infer that specific developmental pathways and tissues exhibit a stronger sensibility to the shortage of such essential metabolite. To further investigate this possibility, we focused our analysis on the effects of a lower dose of *coasy*-MO (1.2 pmol/embryo), associated with a milder reduction of the level of *coasy* mRNA and of CoA content (0.17 vs 0.36 pmol/embryo, P < 0.01, Fig. 2g). At this dose, most of the embryos showed an almost normal morphology at 24 hpf (237/298 out of three experiments). Actually, CNS structures were mildly altered, initial head and heart edema were evident, and somites and tail were quite normal or only partially curved (not shown). At 48 hpf the phenotype appeared more severe: morphants often showed a reduction in size (Supplementary Table S1), but no or modest delay in development as confirmed by the careful observation of the head-trunk angle (HTA) size, that was very similar to that of controls at 48 and 60 hpf^23^. In addition, 81% of *coasy*-MO injected embryos (n = 242/298 out of three independent experiments) had poorly defined brain structures (particularly hindbrain and midbrain) and head edema. The trunk was thinner with loss or perturbation of the chevron-like structure of somites, the tail was often curved or twisted, the ventral fin was reduced or absent and finally, edema was often present in the tail region (Fig. 1f,g). Altogether, our findings seem to indicate that defects in CoA availability severely interfere with neurogenesis, differentiation and with proper establishment of the antero-posterior axis in zebrafish embryos.

To demonstrate the specificity of the observed defects, we tested whether exogenous supply of CoA could prevent the development of the phenotype in embryos exposed to *coasy*-MO. In a dose-curve experiment, CoA added at 5 hpf showed some toxicity (50% of death) at doses higher than 200 μM (not shown), while at 100 μM it was not toxic and induced a relevant increase of CoA concentration in larvae (Fig. 2g). When embryos were injected with 1.2 pmol of *coasy*-MO and supplemented with exogenous 100 μM CoA, a very large percentage of embryos (83%, n = 189/227, at least three independent experiments) showed normal morphology at 24 and 48 hpf (Fig. 2c). Similar results (72%) were obtained in embryos injected with 2.5 pmol of *coasy*-MO (Fig. 2f), thus suggesting that prevention of CoA depletion results in a significant correction of the aberrant phenotype, in particular the antero-posterior axis development and the brain formation.

### Over-expression of human *COASY* mRNA re-establish normal development

**Figure 3:**
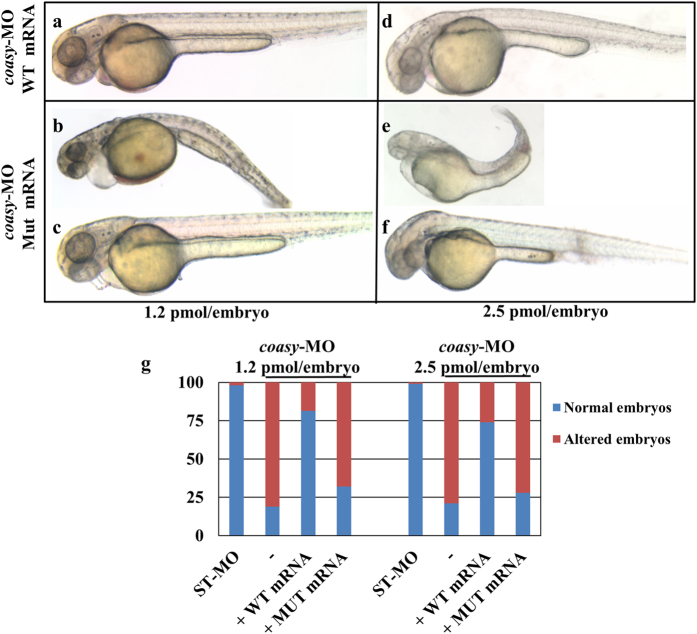
Rescue of *coasy* knock-down by injection of human mRNA. Morphological analysis of morphants co-injected with human COASY mRNA, either wild type (**a,d**) or mutated (**b,c,e,f**) at 48 hpf. Morpholinos were used at either 1.2 (**a–c**) or 2.5 pmol/embryo (**d–f**). Images are from a representative experiment out of four independently performed. The graph in G shows the total distribution of the different phenotypes observed in injected embryos (4 experiments).

To further confirm the specificity of the observed morphological changes we injected *coasy-*MO (1.2 and 2.5 pmol/embryo) together with the human *COASY* mRNA (200 ng), either wild-type or carrying the c.1495 C > T missense mutation identified in two COASY protein-associated neurodegeneration (CoPAN) patients^8^,^9^. The co-injection of the wild-type mRNA fully re-established the normal development of most embryos in both experimental conditions (81.5% n = 72/89 for 1.2 pmol/embryo, and 74% n = 64/86 for 2.5 pmol/embryo) (Fig. 3a). Most of the embryos co-injected with the mutant *COASY* mRNA showed an aberrant phenotype, only partially overlapping with that induced by *coasy*-MO by itself (68% n = 101/148 and 72% n = 89/124 for 1.2 and 2.5 pmol/embryo respectively, Fig. 3b). Interestingly, 32% and 28% of the embryos showed a normal phenotype as opposed to 19% and 21% observed in embryos injected with *coasy*-MO alone. The difference observed at 1.2 pmol/embryo was statistically significant (P < 0.01), suggesting a residual biological function of the over-expressed mutant COASY protein. Altogether these results confirmed the specific relationship between the absence of *Coasy* enzymatic activity and the aberrant development observed in *coasy*-morphants.

### Knock-down of *coasy* and brain development

**Figure 4:**
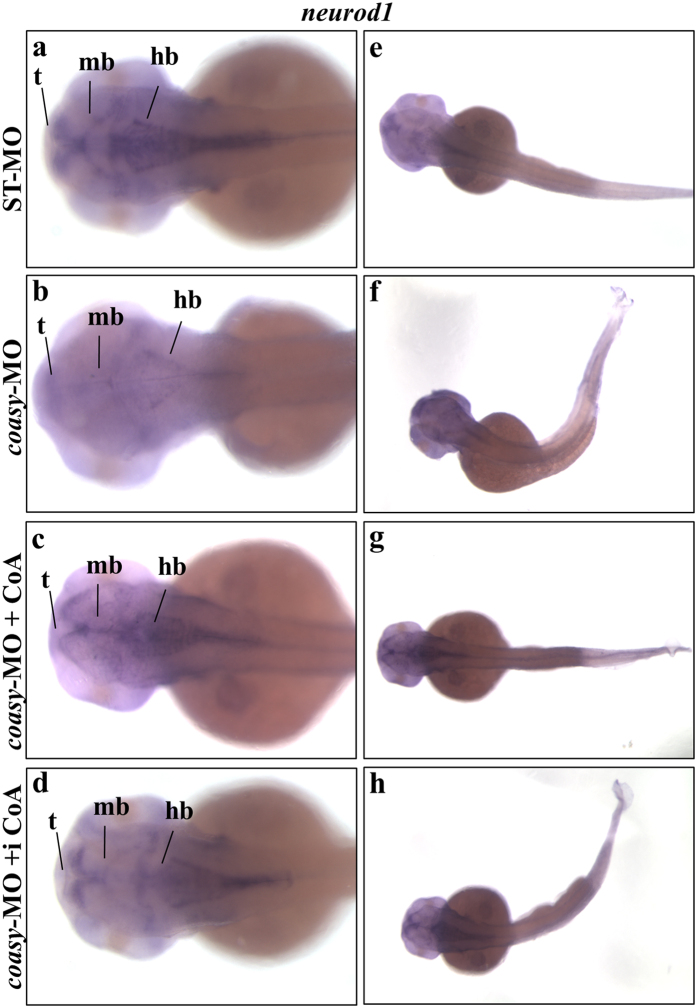
Analysis of *neurod1* neural marker in coasy-morphants. Representative images of WISH analyses of *neurod1* expression in *coasy* morphants at 48 hpf. Dorsal views of ST-MO- (**a,e**) and *coasy*-MO-injected embryos (**b–h**), eventually exposed to CoA in fish water (**c,g**) or injected with CoA in the brain ventricle at 24 hpf (**d,h**). For each panel, results of one representative experiment with at least 40 embryos out of two independent replicates are shown. Abbreviations: d, diencephalon; tg, tegmentum; t, telencephalon; hb, hindbrain; hbn, hindbrain neurons; llg, lateral line ganglia; mb, midbrain; snc, spinal cord neurons.

Down-regulation of *pank2* in zebrafish embryos resulted in significant perturbation of CNS and vasculature structures^13^. We were interested in verifying the possible overlap between these two types of morphants that affect the same biochemical pathway. As shown in Figs 1 and 2, the injection of *coasy*-MO at 1.2 and 2.5 pmol/embryo caused evident alteration of brain morphology. We further investigated this aspect by performing WISH with different neuro-specific riboprobes. Firstly, we analyzed *neurod1* expression*. Neurod1* gene is specifically expressed during central nervous system development in zebrafish and is a key differentiation factor during neurogenesis; it is also involved in the determination of neural subtypes in the ganglia^24^. At 48 hpf the expression of *neurod1* is more specifically localized at tegmentum, diencephalon, hindbrain and lateral line gaglia^25^,^26^. The vast majority of *coasy* morphants (85%, n = 45/53) showed altered *neurod1* expression. The main anterior structures such as telencephalon and midbrain-hindbrain boundary as well as the lateral line ganglia were reduced in size and less intensively decorated by the specific probe as compared to controls (Fig. 4a,b), suggesting an overall reduction in the amount of *neurod1* transcript in these neural regions. As expected, treatment with 100 μM CoA prevented the appearance of the altered phenotype (Fig. 4c). Interestingly, also the direct injection of CoA (0.5 pmol/embryo) in the brain ventricle at 24 hpf lead to a normal expression of *neurod1* (Fig. 4d). This was not associated with a full recovery of the trunk morphology (Fig. 4e–h), suggesting that the depletion of CoA alters larval development by a cell-autonomous mechanism.

**Figure 5:**
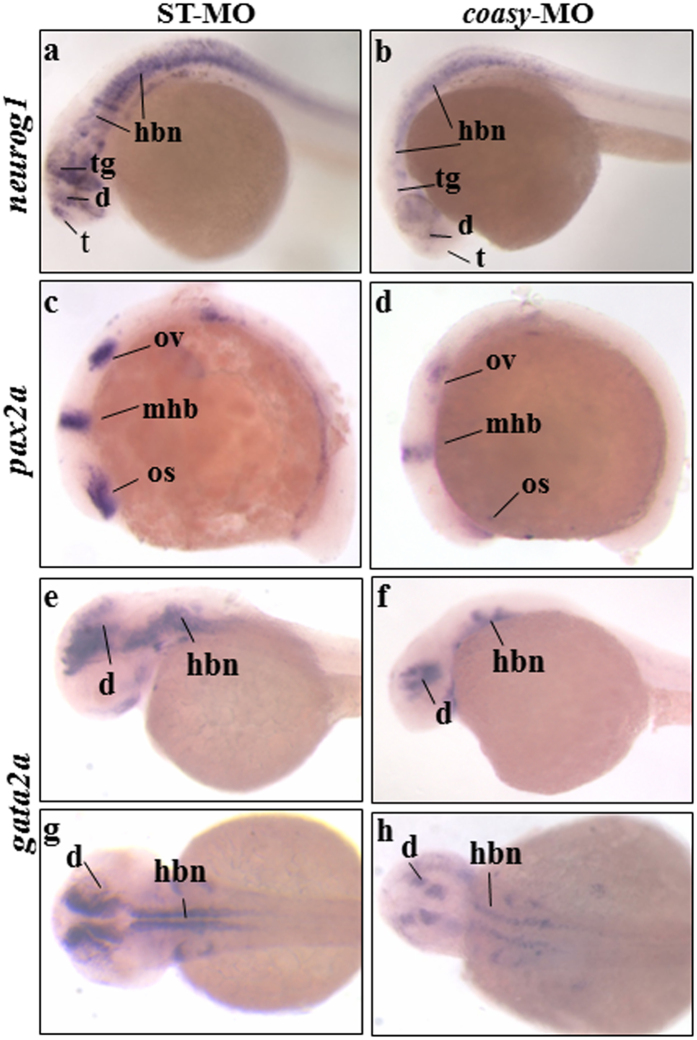
Analysis of *neurog1*, *pax2a* and *gata2a* neural markers in *coasy* morphants. Representative images of WISH analyses with *neurog1, pax2a* and *gata2a* riboprobes. *neurog1* in ST-MO- (**a**), and *coasy*-MO-injected embryos (**b**) at 24 hpf, lateral views. *Pax2a* expression in ST-MO- (**c**), and *coasy*-MO-injected embryos (**d**) at 15 hpf, lateral views. G*ata2a* expression in ST-MO- (**e,f**), and *coasy*-MO-injected embryos (**g,h**) at 17 hpf (**e,f** lateral views and **g,h** dorsal views). Results are from one representative experiment with at least 40 embryos out of two independent replicates. Abbreviations: d, diencephalon; hb, hindbrain; mhb, midbrain-hindbrain boundary; os optic stalk; ov, otic vesiscle; pd, pronephritic ducts.

To corroborate the results obtained with *neurod1* WISH, we investigated the expression of other neural markers. The onset of neurogenesis in the zebrafish neural plate becomes apparent during late gastrulation by the expression of specific proneural genes^27^. *Neurog1* is among the first of these proneural transcription factors expressed in restricted proneural cell clusters^28^,^29^. In addition, *ngn1* is an upstream regulator of *neurod1*^24^, initially expressed in the neural plate and detectable at later stages in tegmentum, dorsal diencephalon, posterior midbrain, midbrain-hindbrain boundary (mhb), hindbrain area, optic stalk and spinal cord. At 24 hpf we observed a strong down-regulation of *neurog1* staining in anterior brain areas (tegmentum, diencephalon, telencephalon) of most *coasy*-MO-injected embryos (n = 51/62, two microinjections) (Fig. 5a,b). Down-regulation of this transcription factor was also evident in hindbrain and spinal cord neurons.

Given the brain structural alteration observed in morphants, it was of interest to explore mechanisms that maintain and refine regional identity in brain during development, that is, the formation of local boundaries between regions such as hindbrain and midbrain. These boundaries often co-localize with local organizing centers within the neural tube mediating specific positional information along the antero-posterior (AP) axis^30^. *Pax2a* gene encodes a paired-box transcription factor involved in this mechanism^31^,^32^. We analyzed the expression of *pax2a* in *coasy*-morphants at early stages of development (12 somites) and found it to be significantly reduced at the mhb (n = 37/47) (Fig. 5c d). Interestingly, the signal was also less intense in the pronephric duct formation (not shown), suggesting an interference also with lateral and ventral mesoderm development. Finally, we analyzed the expression of *gata2a,* a transcription factor required for vascular development and circulation in zebrafish^33^. Three independent enhancers drive the specific expression of *gata2a* in blood precursors, EVL, and CNS of zebrafish embryos^34^. At 48 hpf, the *coasy*-morphants (n = 34/40) showed marked reduction of *gata2a* specific signal in diencephalon and hindbrain when compared to controls (Fig. 5e,h).

Altogether, our WISH analysis by neural-specific riboprobes showed that even a mild reduction of *coasy* mRNA levels perturbs the expression of different proneural transcription factors thereby limiting normal development of many brain structures. Even though some brain regions appeared to be similarly affected both by *pank2* and *coasy* down-regulation, the extent of perturbation induced by reduced levels of *coasy* was significantly larger.

### Knock-down of *coasy* causes defects in vascular development

**Figure 6:**
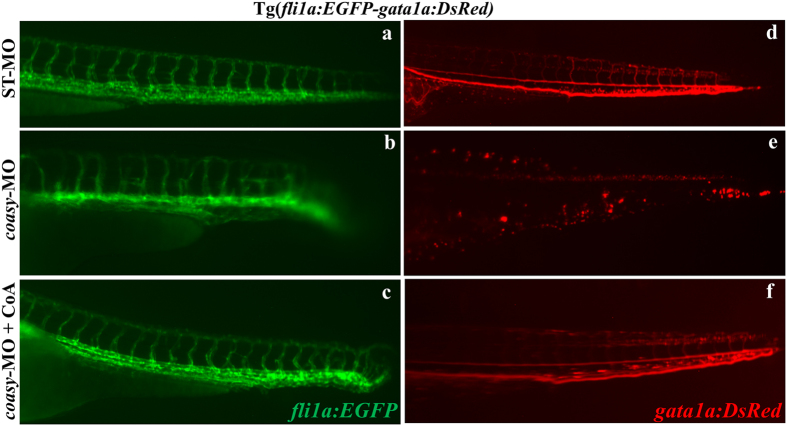
Effects of *coasy*-MO microinjection in the transgenic line Tg(*fli1a:EGFP- gata1a:DsRed*). Lateral views of the trunk/tail regions of Tg(*fli1a:EGFP-gata1a:DsRed*) embryos injected with either ST-MO (**a,d**) or *coasy*-MO (**b,c,e,f**) at 1.2 pmol/embryo, eventually exposed to CoA in fish water (**c,f**) and analyzed at 40 hpf. *fli1a* (EGFP) marks vascular development, and *gata-1* (DsRed) marks red blood cells. In *coasy* morphants compromission of the vascular system and particularly of the intersegmental vessels is evident (**c,d**) and associated with reduction in the number of blood cell (**e,f**). Results are from one representative experiment with at least 80 embryos out of three independent replicates.

Since our previous findings revealed that *pank2* down-regulation affected the formation of the normal vasculature, we extended our analysis by investigating the requirement for *coasy* during vascular system development. For this purpose, we used the transgenic line Tg(*fli1a:EGFP-gata1a:DsRed*). Transgenic embryos were injected with 1.2 pmol/embryo of ST-MO or *coasy-*MO and analyzed at 30 hpf (not shown) and 40 hpf at the fluorescence microscope. The vascular system was severely compromised and the intersegmental vessels were not well formed in *coasy*-MO injected embryos (n = 65/78). The addition of CoA to fish water efficiently rescued the aberrant phenotype (Fig. 6a and c). This phenotype was confirmed also by *in situ* hybridization with the *ve-cadherin*^35^ riboprobe (not shown) and is clearly reminiscent of the one observed in *pank2* morphants. Interestingly, the same transgenic line allowed us to document that *coasy* morphants had a lower number of red cells when compared to controls and this was associated with signals of reduced blood flow (Fig. 6 d-f). These results suggest that *coasy* function is required for normal development of ventral mesoderm and/or its derivatives.

### Knock-down of *coasy* alters *no tail* and *myod1*, but not *vmhc* expression

**Figure 7:**
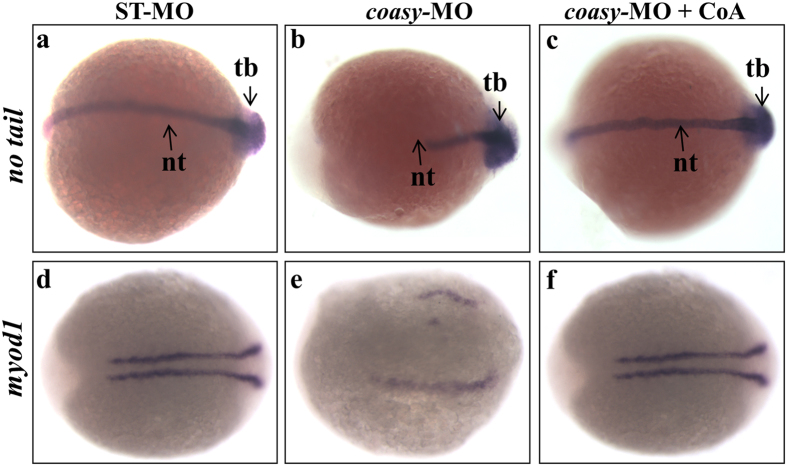
Analysis of *no tail* and *myod1* expression in *coasy*-morphants. Representative dorsal view images of WISH analyses of *no tail* (at 17 hpf) and *myod1* (at 16 hpf) expression in ST-MO- (**a,d**) and *coasy*-MO-injected embryos (**b,c,e,f**), eventually exposed to CoA in fish water (**c,f**). Results are from one representative experiment with about 80 embryos out of two independent replicates.

To study whether *coasy* down-regulation interferes with cellular fate in the dorso-ventral patterning, we evaluated the expression of different markers either involved in or related to this process. *T-box* genes are transcription factors that pattern the vertebrate mesoderm^36^,^37^. *No tail* is one of such genes. In *no tail* mutant embryos, the segmentation of paraxial mesoderm into somites occurs normally, but somite patterning at later stages is abnormal and somites are block-shaped rather than chevron-shaped. The notochord is aberrant and not formed and results in a dramatic shortening of the body axis. We investigated the expression of *no tail* in embryos injected with 1.2 pmol/embryo of *coasy-*MO at 16 somites (17 hpf). As shown in Fig. 7 at 17 hpf structures such as notochord and tail bud are strongly reduced in a large percentage (84% n = 67/80) of *coasy*-morphants compared to ST-MO-injected embryos. The morphological analysis documented that *coasy*-MO injection induced alteration of somite development (Fig. 1). To analyze in deeper detail this feature, we hybridized morphants with a probe for *myod1*, a gene required for musculature differentiation, morphogenesis and somite formation. *Myod1* expression was reduced and indicative of structurally perturbed somites in morphants (76% n = 59/78) as compared to controls at 16 hpf (Fig. 7d,e). Both *no tail* and *myod1* expression were normal when morphants were treated with exogenous CoA (Fig. 7c,f). To analyze whether any structures of mesodermal origin was affected by *coasy* knock-down, we investigated also the expression of ventricular myosin heavy chain (*vmhc*), a cardiac marker, in ST-MO and *coasy*-MO injected embryos at 48 hpf. No significant difference in the expression level of *vmhc* was evident when morphants were compared to control embryos (Supplementary Fig. S6).

### Knock-down of *coasy* reduces *bmp* activity

**Figure 8:**
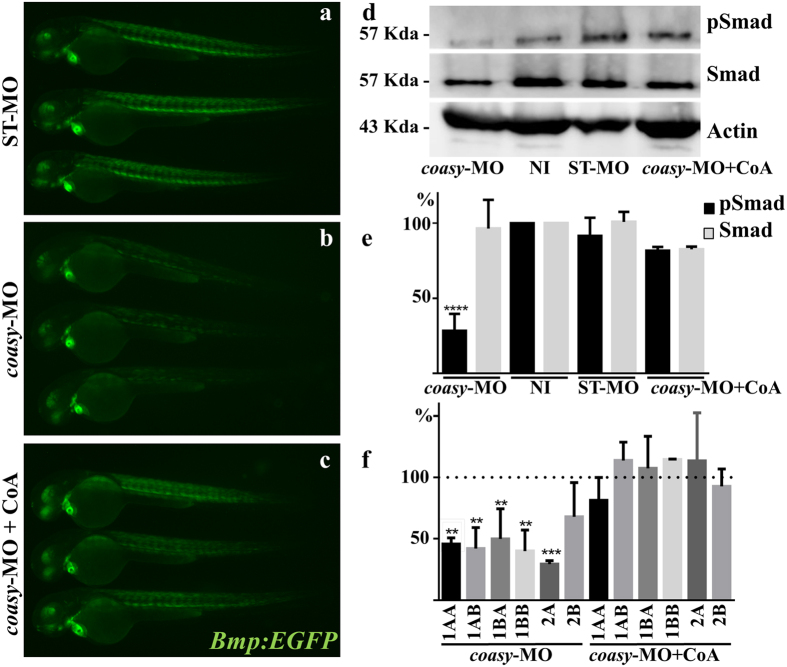
Analysis of Bmp activity in *coasy*-morphants. Representative images of Tg(*Bmp:EGFP*) embryos injected with ST-MO (**a**) or *coasy*-MO (1.2 pmol/embryo, **b** and **c**), eventually exposed to CoA in fish water (**c**), at 60 hpf. A net reduction of the fluorescence intensity is evident in *coasy*-morphants. Results are from one representative experiment with at least 25 embryos out of two independent replicates. (**d**) Western blotting analysis of the amount of phosporylated Smad-5, total Smad, and Actin in embryos injected with *coasy*-MO (±CoA) or ST-MO (1.2 pmol/embryo), and not-injected (NI). Full size images of the gels are shown in Supplementary Fig. S7) The graph shows the quantitative analysis of the immunoblotting. (**f** ) Real-time RT-PCR quantification of different Bmp receptor mRNAs in morphants at 48 hpf, eventually treated with CoA in fish water. Mean values are form three independent experiments and expressed as percentage of the value in ST-MO injected embryos.

The Bmp signaling cascade plays a central role in dorso-ventral patterning of zebrafish embryo^21^,^38^. Different maternal and zygotic factors concur to establish a signaling gradient so that high levels of Bmp induce the development of most ventral mesodermal derivatives such as posterior tail, somites and blood^39^, whereas low levels are required for dorsal fates. Given the dorsalized phenotype observed in morphants both at morphological and functional level, we investigated the activity of this signaling pathway by microinjection of *coasy*-MO (1.2 pmol/embryo) in the transgenic line Tg(*Bmp:EGFP*), which reports Smad-mediated Bmp signaling in embryos and adults. This line expresses enhanced green fluorescent protein (EGFP), under the Bmp Response Element^40^,^41^. Down-regulation of *coasy* resulted in a significant decrease of the fluorescent signal at 60 hpf (Fig. 8 a and b), but the decrease was already evident at 30 hpf (Supplementary Fig. S7). The reduction was particularly evident in somites and eye, whereas less pronounced or absent in the heart. Treatment of embryos with CoA prevented the decrease of Bmp signaling (Fig. 8 c) either when added to fish water (100 μM) or directly injected (0.5 pmol/embryo) in the craniofacial region of morphants at 24 hpf (Supplementary Fig. S7). To confirm the result, we analyzed the phosphorylation of Smad5 protein (pSmad5), a downstream effector of Bmp-receptor activation in the same embryos. The immunoblot analysis showed a robust reduction in the intensity of pSmad5 band (about 70%, P < 0.0001) in *coasy*-MO-injected embryos as compared to not-injected embryos (Fig. 8d and e). No decrease in pSmad phosphorylation was evident in morphants exposed to 100 μM CoA in the water. In order to dissect the mechanism underlining this phenomenon, we quantified mRNA levels of ligands and receptors potentially involved in activation of the signaling cascade. While no difference was evident for Bmp-2A, Bmp-2B and Bmp-4 expression levels (Supplementary Fig. S9), a robust reduction was present for all the Bmp receptor isoforms we investigated, with the exception of BmpR-2B (Fig. 8f). On the basis of the fundamental involvement of Bmp signaling during vertebrate development as well as in adult tissues homeostasis, the results may be of extreme relevance for the interpretation of the phenotype of *coasy*-morphants and eventually indicates a new perspective for the analysis of CoPAN pathogenesis.

### Knock-down of *coasy* induces cell death

Given the central role of CoA in energetic metabolism and the clear lethality associated with the strong reduction in *coasy* expression, we evaluated cell death events induced by the lower dose of *coasy*-MO using acridine orange staining^42^ (Supplementary Fig. S10). Florescence imaging showed a clear increase of the staining intensity in morphants, particularly evident in the head and tail regions. The quantitative analysis performed on lysates from pooled embryos documented a 2.5-fold increase in *coasy*-MO versus control embryos.

## Discussion

NBIA is a set of clinically and genetically heterogeneous disorders, characterized by progressive neurodegeneration and accumulation of iron in the brain, most commonly in the basal ganglia. In the recent years an intense effort to identify the genetic basis of this type of diseases led to the discovery of mutations in at least 10 different genes^5^ and opened the way to the study of the molecular mechanisms underlining the neurodegenerative processes. Among this set of genes, two codes for enzymes catalyzing limiting steps in CoA biosynthesis: PANK2, linked to PKAN^6^ controls the phosphorylation of pantothenate to 4′-PPA while COASY, linked to CoPAN^8^,^9^, mediates the two final steps of the pathway, from 4′-PP to dephospho-CoA and finally to CoA. This drove the attention to the role played by this metabolite in neural development and functioning^3^. CoA and its derivatives are essential for many biochemical processes, such as energy production, lipid metabolism and heme synthesis, involved both in neurodegeneration and iron metabolism^43^. Quite unexpectedly, defects in CoA production results in disorders with prominent, if not exclusive, involvement of the CNS and particularly of the nuclei involved in movement control. This is surely one of the central aspects of the pathogenesis of these disorders that remains poorly understood, together with the connection with brain iron homeostasis and the very early onset of symptoms.

We tried to get more insights into the pathogenesis of these disorders by performing morpho-functional studies of *pank2* and *coasy* genes during the early phases of zebrafish development. *Pank2* mRNA was detected at highest levels in the CNS of fish embryos and larvae; the injection of a specific morpholino leads to a significant reduction of *pank2* expression associated with evident perturbation of brain and vasculature development. Particularly affected was the telencephalon, presumably containing nuclei corresponding to the human globus pallidum^44^, where the expression of the neuronal differentiation marker *neurod1* was dramatically reduced. This indicated an essential role for *pank2* in the development and differentiation of specific sets of neural network, not compensated by other *pank* isoforms, but strongly linked to the production of CoA, as documented by the rescue capability of CoA and pantethine supplemented to the fish water. Interestingly, also the development of the vasculature arborization was perturbed, particularly in the head and the tail, thus suggesting a putative involvement of defective angio- and/or vasculogenesis in the pathogenesis of the human disease. On the basis of these results, we extended our analysis to *coasy* gene. As in most vertebrates, also in *Danio rerio* there is a single *coasy* gene, with high level of identity to the human homolog. It is a maternal gene with stable expression throughout the different stages of zebrafish embryonal development and ubiquitously transcribed in the adult, with higher levels in liver, brain and eye. The apparently complete down-regulation of *coasy* expression obtained by high doses of morpholino (2.5 pmol/embryo) leads to a severe alteration of development with death occurring within 72 hpf. Interestingly a large percentage of embryos showed a phenotype recalling C3 and C4 dorsalized mutants of ventral specifying genes^21^. Together with the alteration of tissues of ventral origin, also the brain was poorly defined. The partial down-regulation of *coasy* expression resulted in a milder phenotype that confirmed the important sensibility of neurogenesis and ventralizing pathways. These phenotypic features were specifically associated with a shortage of CoA availability, as measured by a specific LC-MS/MS method. Indeed, CoA supplementation to fish water or overexpression of the human wild-type *COASY* mRNA could prevent the abnormal phenotype. The injection of CoA in the brain ventricle of morphants allowed the local rescue of *neurod1* expression, but did not seem to be able to efficiently prevent the alteration of mesodermal structures. This allows to infer that decrease of CoA level in each cell is the main trigger of the altered development, but the experiments so far performed cannot exclude possible cross-talks among different tissues.

Interestingly, the co-injection of human mutant mRNA with 1.2 pmol/embryo of coasy-MO ushered in a modest, yet statistically significant prevention of the altered morphology, thus indicating a partial preservation of the biological function of the mutated allele *in vivo*. This is in line with data from Dusi and coauthors^8^ showing loss of activity for the recombinant mutant protein *in vitro*, yet the persistence of CoA levels and *de novo* production in fibroblasts from patients carrying the same Arg499Cys substitution. While showing a modest rescuing capacity, the co-injection of mutant COASY mRNA lead to an aberrant phenotype that often differed from that of *coasy* morphants. Since no changes in morphology were observed when either wild-type or mutant mRNA were injected, the interpretation of this results is rather puzzling and will require further studies.

The *in situ* hybridization with neural specific probes confirmed the results of the morphological analysis on *coasy* morphants. Reduction in size and poor definition of brain structures was clearly associated with marked decrease of *neurogenin1, neurod1, pax2a* and *gata2a* signal. Contrary to what observed with *pank2* suppression, all investigated brain regions and structures were affected, suggesting a more generalized perturbation of neural differentiation. When we evaluated the development of the vasculature by a specific probe (*ve-cadherin*) and in a *fli1a* trangenic line, we detected an altered vasculature structure, particularly in brain and trunk, with reduction in ISV formation associated to decreased red cells and blood flow. These findings overlap with the phenotype observed in *pank2* morphants and highlights the pivotal role of adequate levels of CoA for normal vasculogenesis and angiogenesis. There is no proof yet that this holds true in the human pathology, but it could be of interest to investigate this aspect in mammalian experimental models of PKAN and CoPAN.

The shortage of CoA dramatically affected the development of zebrafish, with appearance of phenotypes with graded levels of dorsalization. This could be in part explained with the significant down-regulation of the Bmp signalling pathway, as clearly evidenced by the reduced fluorescent signal and Smad 5 phosphorilation observed in the Bmp-reporter transgenic line injected with *coasy*-MO (Fig. 8). BMPs comprise a large family of secreted signaling factors belonging to the transforming growth factor-beta (TGF-β) superfamily and are expressed in both developing and adult tissues. In zebrafish they are necessary for specification of posterior tail and ventral fates such as somites and blood^38^,^45^. The reduction in Bmp transcriptional activity we documented was obtained with injection of 1.2 pmol/embryo of *coasy*-MO and can explain the alteration observed in morphants, but could be more severe at higher morpholino doses, when the morphology more clearly resembled that of dorsalized mutants. While we did not observe significant changes in the mRNA level of the main Bmp isoforms, we found lower levels of Bmp-receptors expression, which could explain the initial result. We are not aware of any mechanism directly linking CoA metabolism and Bmp-receptors expression, and further work is necessary to better understand this result. Other mechanisms could contribute to the suppression of Bmp signaling. Interestingly, Miyares and coauthors^46^ documented the requirement for long-chain acyl-CoA synthetase 4a (*Acsl4a*) for proper Bmp signaling and patterning of the zebrafish dorso-ventral axis. G.

Indeed, gene expression patterns (*gata2a, pax2a, myod1*) and morphological features (dorsalization) of *ascl4a* morphants overlap with those of *coasy* ones. Ascl4a converts long chain-polyunsaturated fatty acids (LC-PUFA) to acyl-CoA that are fundamental for lipid metabolism and cell signaling. Mutations in the human ACSL4 ortholog are associated with X-linked mental retardation^47^ and the *Drosophila* hypomorphs show defects in segmentation^48^ and development of CNS, with reduced number of glial and neural cells in the brain^49^, as well as diminished synaptic growth and function^50^,^51^. These features could be explained, at least in part, by the reduction in Bmp activation observed both in zebrafish and in *Drosophila*. The enzyme can actually control the levels of Smad transcription factors by blocking the inhibiting activity of glycogen synthase kinase 3 and p38 mitogen-activated protein kinase, as shown in zebrafish^46^, but can also affect vesicles trafficking and membrane recycling, and hence the availability of Bmp receptor, as observed in *Drosophila*^51^. The reduction of CoA levels induced by down-regulation of Coasy can surely have an impact on LC-PUFA activation process and therefore affect Bmp signaling.

Perturbation of the ventral-to-dorsal Bmp gradient can be directly linked with many of the morphological and functional defects observed in *coasy* morphants. The decreased expression of makers such as *gata2*, *myod1* and *pax2a* in tissues of mesodermal origin as well as the reduction in blood cells and ISVs formation can be ascribed to a defective Bmp signaling.

Restriction of Bmp expression to the ventral part of the embryo by the onset of gastrulation is instrumental for proper ectoderm neutralization^39^,^52^. Yet Bmp activity persists in the CNS at later stages and exerts a fundamental role in neurogenesis and neural differentiation, as documented in the retina^53^ or in the inner ear^54^. This is indirectly confirmed by the spatial analysis of EGFP expression in the Bmp-reporter line^41^ that documented a clear signal in eye and other brain regions (mhb) from 24 to 72 hpf. Hence, the reduction of Bmp expression induced by *coasy* down-regulation may concur to the insurgence of the brain structural and functional defects observed in morphants.

We did not explore iron homeostasis in *pank2*- and *coasy*-morphants, and zebrafish embryos are not the most suitable system to investigate iron accumulation, but it is interesting to observe that Bmp-signaling is the main regulator of hepcidin expression, the master regulator of systemic and cerebral iron balance^55^. Our findings may indicate a new perspective to investigate the mechanisms underlining brain iron accumulation in these disorders.

Given the central role of CoA in cellular metabolism and energetic processes, it is possible that other mechanisms and signaling pathways contribute to the observed phenotype, but the analysis performed at lower doses of morpholino and with milder reduction in *coasy* expression suggests a high sensibility of the Bmp signaling cascade to the shortage of CoA.

It is possible that perturbed lipid metabolism, including LC-PUFA, and membrane homeostasis is one of the immediate consequences of limited CoA availability. This seems to be a unifying theme for many types of NBIA disorders, and particularly for CoPAN, PKAN, PLAN, FAHN and MPAN, which are characterized by alteration of phospholipids and sphingolipids metabolism, membrane remodeling and mitochondrial morphology and functioning^56^. Further investigation of the possible connection between lipid dyshomeostasis and Bmp signaling cascade may provide relevant insight into the pathogenesis of these disorders and the specific neuronal involvement.

## Materials and Methods

### Zebrafish maintenance and stocks

Zebrafish were maintained and used following protocols approved by the Local Committee (*Organismo Per il Benessere Animale*–Committee for Animal Health) (No. 211B5-10) and in accordance with the Italian and European regulations on animal use. Breeding, and staging were performed as previously described^23^. Beginning from 24 hpf, embryos were cultured in fish water containing 0.003% 1-phenyl-2-thiouera (PTU) to prevent pigmentation. The following strains were used: the wild type AB, and the transgenics Tg(*fli1a:EGFP;gata1a:DsRed*)^57^ and *Tg(BRE-AAVmlp:EGFP)*^*mw29*^ [hereafter Tg(*Bmp:EGFP*)]^41^.

### Bioinformatic analysis

Zebrafish genomic sequences were analyzed using the University of California Santa Cruz (UCSC) Genome Browser (http://genome.ucsc.edu/) on the Zv10 (Sept 2014) *Danio rerio* assembly and the Ensembl zebrafish genome database (http://www.ensembl.org/Danio_rerio/Info/Index).

Synteny analysis was achieved using the Synteny Database^19^. Nucleotide and amino acid sequences were compared to the non-redundant sequences present at the NCBI (National Center for Biotechnology Information) using the Basic Local Alignment Search Tool (BLAST)^58^. Multiple sequences alignment was performed using the MUSCLE algorithm^59^.

### RNA extraction and Real Time RT-PCR

Total RNA was extracted from 30 embryos for each different developmental stage analyzed, frozen in liquid nitrogen, using TRI-Reagent (Sigma) according to manufacturer’s protocol. For tissues dissection, the adult fishes were euthanized by an excess of ethyl 3-aminobenzoate methanesulfonate salt solution (Sigma). RNA was quantified using the My Spect spectrophotometer (Biomed) and controlled by electrophoretic separation on a 1% TAE-agarose gel. 1.0 μg of total RNA was retro-transcribed to cDNA using Im-Prom Reverse Transcriptase (Promega) and oligo (dT) primers following the manufacturer’s protocol. Primers were designed by the PrimerQuest and Real Time PCR Tool from IDT (Supplementary Table S2). Real-Time PCR was performed using the Eco-Illumina system. Reactions were performed in a 10 μl volume, with 0.5 μM of each primer (sequences in Supplementary Table S2), 5 μl of Syber Green Master Mix (Biorad) and 20 ng of cDNA. The amplification profile consisted of a denaturation program (95 °C for 1 min), 40 cycles of two steps amplification (95 °C for 15s and 60 °C for 30s) followed by a melting cycle. Each reaction was performed in triplicate and when required *actin beta 1* was used as a reference gene. Relative levels of expression were calculated by the ΔΔCT method.

### Riboprobes synthesis and whole-mount *in situ* hybridization

Total RNA was extracted from a pool of 2-cell stage embryos using TRI-Reagent (Sigma) according to manufacturer’s protocol. RNA was quantified using the My Spect spectrophotometer (Biomed) and controlled by electrophoretic separation on a 1% TAE-agarose gel. 1.0 μg of total RNA was retro-transcribed to cDNA using Im-Prom reverse transcriptase (Promega) and oligo(dT) primers following the manufacturer’s protocol. To synthesize the riboprobes for the detection of zebrafish *coasy* transcripts, we amplified a specific region by PCR using as template the cDNA from the 2-cell stage and oligonucleotide primers (P3 and P4, Table S2). The amplification conditions were the following: 2 min at 95 °C, 30 cycles at 94 °C for 15 sec, 60 °C for 30 sec, 72 °C for 1 min, followed by final extension at 72 °C for 5 min. The PCR product was subcloned with the pGEM-T-Easy system (Promega), and verified for sequence and orientation of the inserts. Antisense and sense RNA probes were obtained by *in vitro* transcription of the cloned cDNAs with T7 or SP6 RNA polymerase, using a digoxigenin labeling mixture according to manufacturer’s instructions (Roche). Whole-mount *in situ* hybridization (WISH) was performed according to a standard method^60^. Briefly, embryos and larvae were collected, dechorionated and incubated at 28 °C at different stages. Embryos were fixed overnight in 4% paraformaldehyde (PFA) at 4 °C, dehydrated through an ascending methanol series and stored at −20 °C. After treatment with proteinase K (10 μg/ml, Roche), the embryos were hybridized overnight at 68 °C with DIG-labeled antisense or sense RNA probes (400 ng). Embryos were washed with ascending scale of Hybe Wash/PBS and SSC/PBS, then incubated with anti-DIG antibody conjugated with alkaline phospatase over night at 4 °C. The staining was performed with NBT/BCIP (blue staining solution, Roche) alkaline phosphatase substrates. When different type of probes (sense vs antiense) or fish (injected vs not-injected) had to be compared, all incubations were carried out at the same time, at the same probe concentration and, when required, with the same reagents and solutions. WISH images were taken with a Leica MZ16F stereomicroscope equipped with DFC 480 digital camera and LAS Leica Imaging software (Leica). Magnification: 50X, 63X, and 80X.

### Morpholino injections

Injections were carried out on one/two-cell-stage embryos (with Eppendorf FemtoJet Micromanipulator 5171); the dye tracer phenol-red was co-injected as a control. To repress *coasy* mRNA translation we designed a splicing-inhibiting morpholino oligo (*coasy*-MO, Table S2); as a negative control we injected a standard control morpholino oligo (ST-MO) (Gene Tools) which targets a human beta-globin intron (Supplementary Table S2). Morpholinos were injected in Danieau buffer (pH 7.6) as previously described^61^. After microinjection, embryos were incubated in egg water supplemented with 0.003% PTU at 28 °C to prevent pigmentation processes. Embryo development was evaluated at 24 and 48 hpf. RT-PCR experiments were performed on RNA extracted from *coasy*-MO- or ST-MO-injected and wild type embryos at 24 hpf and 48 hpf, with specific primers (P5 and P6, Supplementary Table S2). Control RT-PCR amplification on the same RNAs was carried out with *actin beta 1* primers (A1 and A2, Supplementary Table S2). When indicated, the comparison between morphants and controls was performed by selecting morphants with the mildest phenotype (embryos injected with 1.2 pmol of CoA-MO) and no evident delay in development (head-trunk angle).

### Rescue of phenotype by *COASY* human wild type and mutant mRNA

The pGEM plasmids containing the coding region for human wild type and c.1495 C > T mutant *COASY* allele were kind gifts from Dr. Valeria Tiranti, *Fondazione I.R.C.C.S. Istituto Neurologico Carlo Besta*, Milan. The plasmids were digested with NcoI restriction enzyme and then transcribed using the mMESSAGE mMACHINE *in vitro* Transcription Kit (Life Technologies) according to the manufacturer’s instructions. Dose-response curve experiments were performed in wild type embryos to identify the maximum amount of *coasy* mRNA that did not induce phenotypic alterations. The rescue experiments were carried out by co-injecting *coasy*-MO together with 200 pg/embryo of human wild-type or mutant *COASY* mRNA.

### CoA treatment and LC-MS/MS analysis of CoA

Control embryos and embryos injected with *coasy*-MO at 1.2 and 2.5 pmol were treated with 100 μM CoA in fish water at 5 hpf. At 48 hpf survived embryos were decoryonated, rinsed thoroughly with water and prepared for morphological analysis at the microscope or pooled in groups of 50 and frozen for CoA content analysis. Frozen samples were thawed and 150 μl of 5% perchloric acid (PCA) was immediately added. Samples were homogenized using a pipette, vortexed, sonicated and centrifuged at 18,000 × g for 5 minutes at 4 °C. The injection mixture was filtered through a 0.2 μm PVDF filter. The filtrates were brought to a volume of 0.5 ml with water and 10 μl were injected into the system LC-MS/MS. Calibration curves (100, 10, 1, and 0.1 pmol/μl) were prepared by dilution of the CoA standard (Supplementary Fig. S5). Linearity was determined by least-squares regression. An HPLC 1260 System equipped with an infinity system model 1260 injector and paired with an Agilent Technologies (Waldbronn, Germany) 6460 Triple Quad LC/MS triple quadruple mass spectrometer were used for the quantitative dosage of CoA. Chromatographic separation of CoA was achieved using INERTSIL ODS-3 C18 column (100 × 3 mm × 3μm) coupled with a security guard column. The flow rate was 0.3 mL/min with mobile phase of 0.01% Formic Acid in water (A) and Methanol (B). The column was equilibrated with 2% B for 3 min, a linear gradient to 95% B was applied over 12 min. The total run time was 15 min per sample. The optimal source parameters were: source temperature: 300 °C, drying gas flow: 12 L/min, drying gas temp: 350 °C, nebulizer pressure: 40 psi, capillary voltage: 3,500 V. Mass spectrometry was performed in the positive ion mode, monitoring the MS/MS transitions m/z 768.1 → 428.2 and 768.1 → 261.

### Staining with Acridine Orange

Acridine orange (AO) staining is a nucleic acid selective metachromatic stain technique that can identify cell death. For AO staining, embryos at 48 hpf were incubated for 30 min in fish water containing acridine orange at 10 mg/l. Embryos were then rinsed three times in fish water, anesthetized with tricaine, and quickly imaged by epifluorescent microscopy. Pools of 10 embryos were than lysed in 100% ethanol and fluorescence was measured at λex 490 nm/λem 525 nm with a microplate reader (Ensight Perkin Elmer).

### Immunoblotting

20 embryos for each condition were manually dechorionated. Proteins were extracted by homogenizing embryos in buffer (200 mM Tris/HCl, 100 mM NaCl, 1 mM EDTA, 0.5% NP-40, 10% Glycerol, 1 mM NaFl and sodium orthovanadate) on ice and quantified by a standard bichinonic acid method. 80 μg of total protein extract were then separated on two 10% polyacrylamide gels, one for pSmad and the other for total SMAD detection, transferred on to membranes (GE Amersham^TM^ Hybond^TM^ P 0.45 PVDF). Membranes were cut at the level of the molecular weight of interest, blocked with 2% skim milk in TBST, incubated with the primary antibody diluted in 2% skim milk in TBST (phospho-Smad5, 1:1000, Abcam #ab92698, Smad5, 1.1000, Cell signaling Technology #9517) and then with the anti-rabbit IgG secondary antibody also prepared in 2% skim milk (1:2000). ECL Western blotting detection reagent (Pierce) was used for detection. Images were acquired with Li-Cor Odissey image station, and band intensity quantified by ImageJ software without any modification of the original data. Original images were partially cropped to fit to the final figure.

### Statistical analysis

The data are reported as the sum or as representative of at least three independent experiments with similar results. Statistically significant differences between different types of embryos were calculated by one-way ANOVA analyses; *, ** and *** indicate P < 0.05, <0.01 and <0.001), respectively. The Chi square test was applied to compare frequencies of morphants and normal embryos in different types of samples.

## Additional Information

**How to cite this article**: Khatri, D. *et al*. Down-regulation of *coasy*, the gene associated with NBIA-VI, reduces Bmp signaling, perturbs dorso-ventral patterning and alters neuronal development in zebrafish. *Sci. Rep.* **6**, 37660; doi: 10.1038/srep37660 (2016).

**Publisher's note:** Springer Nature remains neutral with regard to jurisdictional claims in published maps and institutional affiliations.

## Supplementary Material

Supplementary Information
