# Supplementary material for: Down-regulation of coasy, the gene associated with NBIA-VI, reduces Bmp signaling, perturbs dorso-ventral patterning and alters neuronal development in zebrafish
Source: Sci Rep. 2016 Nov 28;6:37660. doi: 10.1038/srep37660 (PMC5124858; doi:10.1038/srep37660)
Supplement: Supplementary Information [file srep37660-s1.pdf]

Down-regulation of *coasy*, the gene associated with NBIA-VI, reduces Bmp signaling, perturbs dorso-ventral patterning and alters neuronal development in zebrafish.

Deepak Khatri[1°], Daniela Zizioli[1°], Natascia Tiso[2], Nicola Facchinello[2], Sara Vezzoli[1], Alessandra Gianoncelli[1], Maurizio Memo[1], Eugenio Monti[1], Giuseppe Borsani[1], Dario Finazzi[1,3*].

Figure S1

A

```
Hsa-COASY      MRTPRLRAQPRGAVYQAPSPPPAPVGLGSMAVFRSGLLVLTTPLASLAPRLASILTSAAR
Dre-Coasy      -----------------------------MSMFSTGILVLTSPLHVLPLRIAPVLTSAAQ
                                            *::* :*:****:**  *. *:*.:*****.

Hsa-COASY      LVNHTLYVHLQPGMSLEGPAQPQSSPVQATFEVLDFITHLYA-GADVHRHLDVRILLTNI
Dre-Coasy      VVERTLYVHLHPGLNLGTGGQVRPVYIPPVVDLCTLISRLYSNAADICGHLDVRVLLTNI
               :*:.******:**:.*   .* ..  : ...::  :*:.**: .**:  *****:*****

Hsa-COASY      RTKSTFLP----PLPTSVQNLAHPPEVVLTDFQTLDGSQYNPVKQQLVRYATSCYSCCPR
Dre-Coasy      RGQSAASSAANGPFPAP-QTLSHSPEVVLTDFPIPDSGQSSLITQCLRKYAGHCYVCTPG
               * :*:  .    *:*:. *.*:*.********   *..* . :.* * .**  ** * *

Hsa-COASY      LASVLLYSDYGIGEVPVEPLDVPLPSTIRPASPVAGSPKQPVRGYYRGAVGGTFDRLHNA
Dre-Coasy      LSSVLLHPQ--LKEV--------------SGDEDRGAQMKPLETFSDVVVGGTFDRLHGA
               *:****:.:  : **              ...   *:  :*:  :   .*********.*

Hsa-COASY      HKVLLSVACILAQEQLVVGVADKDLLKSKLLPELLQPYTERVEHLSEFLVDIKPSLTFDV
Dre-Coasy      HKTLLNISCLMANRRFIIGVCDQELLKNKVLKELIEPYDQRVEKLHDFLNDVKPSLKYDI
               **.**.::*::*: .:::**.*::***.*:* **::** :***:* :** *:****.:*:

Hsa-COASY      IPLLDPYGPAGSDPSLEFLVVSEETYRGGMAINRFRLENDLEELALYQIQLLKDLRHTEN
Dre-Coasy      VPLSDPFGPSITEPELQCIVVSEETRKGGEAVNKRRVQNGLAELVLYEIPLLKDAHRADI
               :** **:**: ::*.*: :****** .** *:*. *::*.* **.**:* **** ..::

Hsa-COASY      EEDKVSSSSFRQRMLGNLLRPPYERPELPTCLYVIGLTGISGSGKSSIAQRLKGLGAFVI
Dre-Coasy      EEEKISSSSLRTRLLGTLLKPPSPELDLPLCPYVIGLTGGSGSGKSSIARRLEDLGAERI
               **:*:****:* *:**.**.**    :** * ******* *********.**:.***  *

Hsa-COASY      DSDHLGHRAYAPGGPAYQPVVEAFGTDILHKDGIINRKVLGSRVFGNKKQLKILTDIMWP
Dre-Coasy      DCDLLGHEAYLPETSAYHRVIQEFGTDILNEDKSINRRVLGGKVFGNQERLKALTDIVWP
               *.* *** ** *  .**: *:: ******::*  ***.***..****::.** ****:**

Hsa-COASY      IIAKLAREEMDRAVAEGKRVCVIDAAVLLEAGWQNLVHEVWTAVIPETEAVRRIVERDGL
Dre-Coasy      EIARLVKKRIDQAKQQGKRVCVVDAAVLLEAGWTHLVHEVWVATIPEEEAVKRIVQRDGV
                **.*..: :*.*  :******:********** :******.*.*** ***.***:***:

Hsa-COASY      SEAAAQSRLQSQMSGQQLVEQSHVVLSTLWEPHITQRQVEKAWALLQKRIPKTHQALD--
Dre-Coasy      KEEDAVRRLKSQWPNAKLIDYANVVLCTLWEPEVTQKQVLKAWSLLQQRIQKRQETRSSL
               .*  *  **:** .. :*:: ::***.***** :**.** ***:***:** * ::: .
```

B

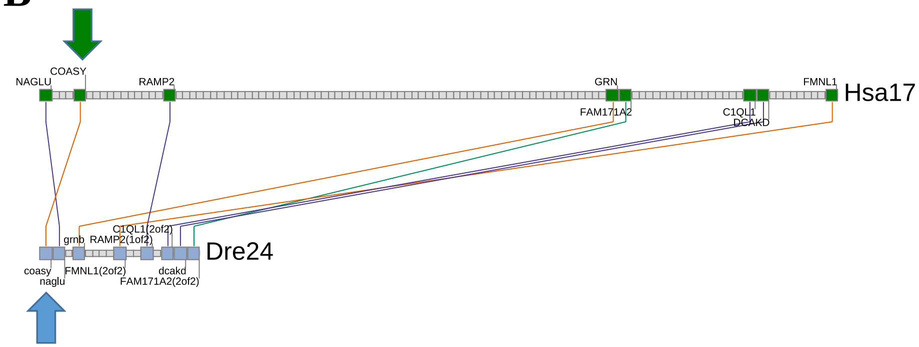

Figure S2

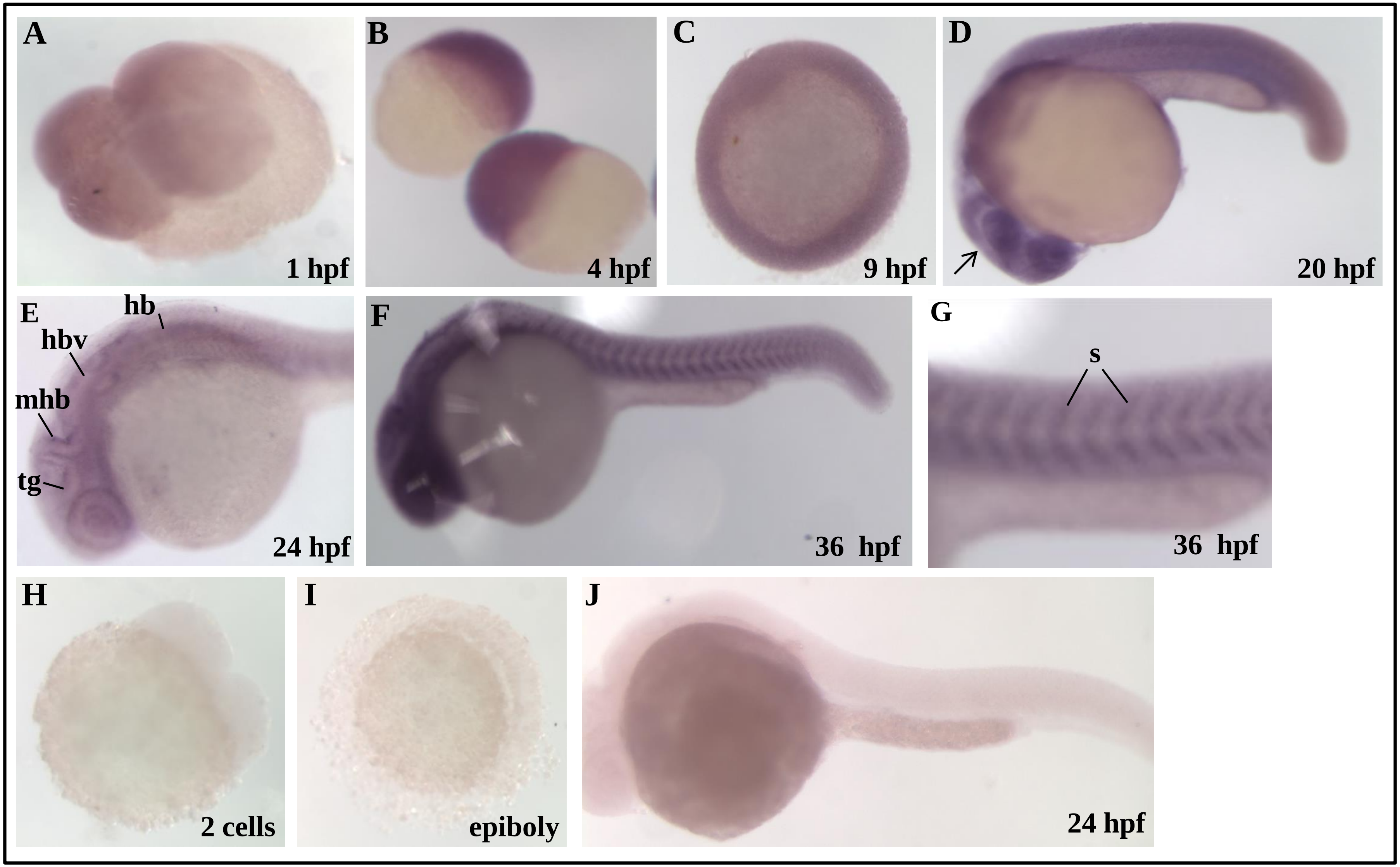
A
1 hpf
B
4 hpf
C
9 hpf
D
20 hpf
E
hb
hbv
mhb
tg
24 hpf
F
36 hpf
G
s
36 hpf
H
2 cells
I
epiboly
J
24 hpf

Figure S3

A

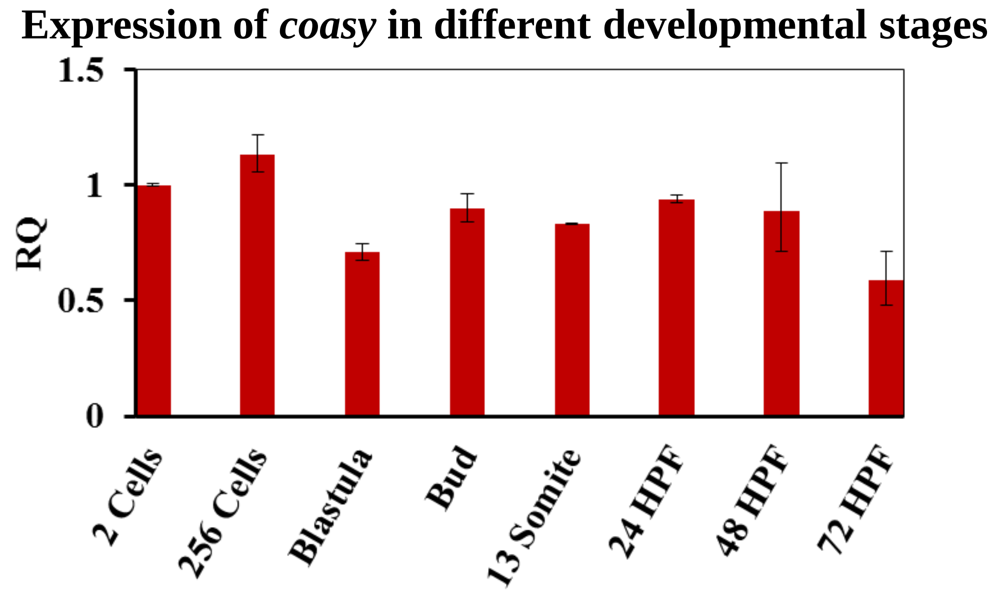
Expression of *coasy* in different developmental stages
1.5
1
0.5
0
RQ
2 Cells
256 Cells
Blastula
Bud
13 Somite
24 HPF
48 HPF
72 HPF

B

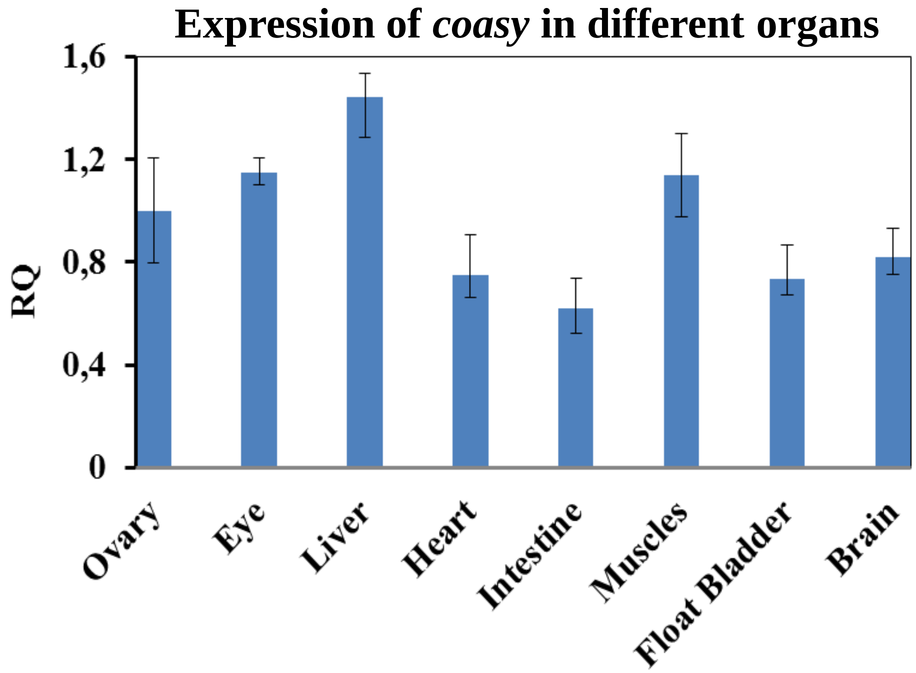
Expression of *coasy* in different organs
1,6
1,2
0,8
0,4
0
RQ
Ovary
Eye
Liver
Heart
Intestine
Muscles
Float Bladder
Brain

Figure S4

A

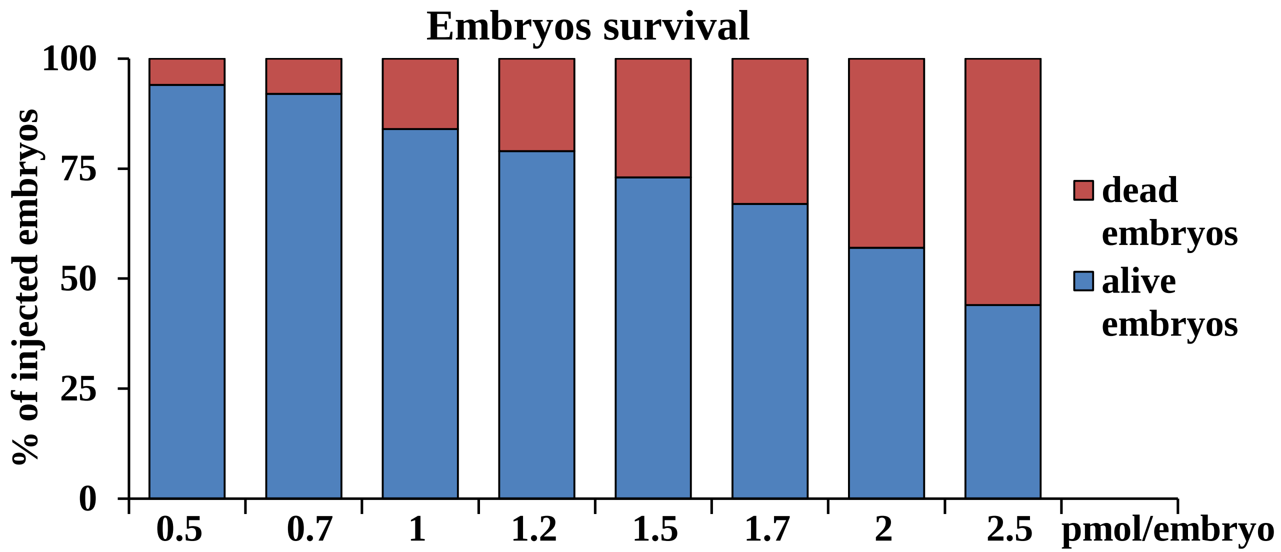
Embryos survival
% of injected embryos
100
75
50
25
0
0.5
0.7
1
1.2
1.5
1.7
2
2.5
pmol/embryo
dead embryos
alive embryos

B

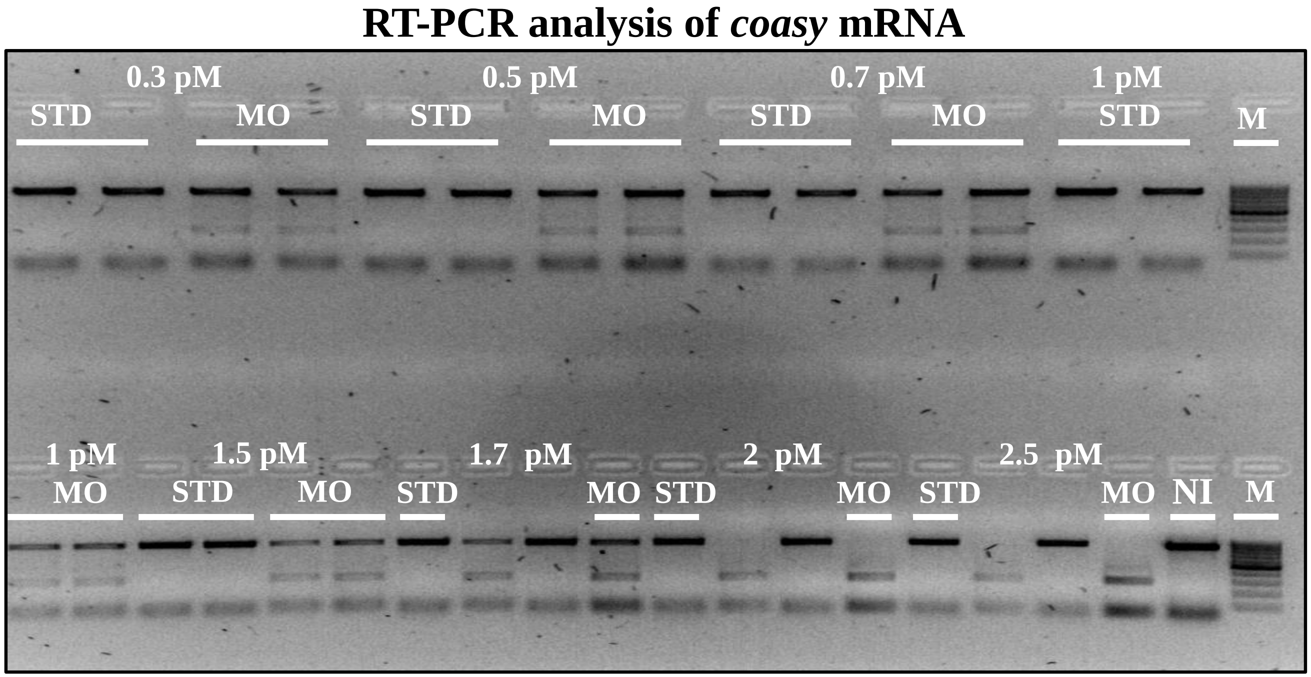
RT-PCR analysis of *coasy* mRNA
0.3 pM
STD
MO
0.5 pM
STD
MO
0.7 pM
STD
MO
1 pM
STD
M
1 pM
MO
1.5 pM
STD
MO
1.7 pM
STD
MO
STD
2 pM
MO
STD
2.5 pM
MO
NI
M

Figure S5

A

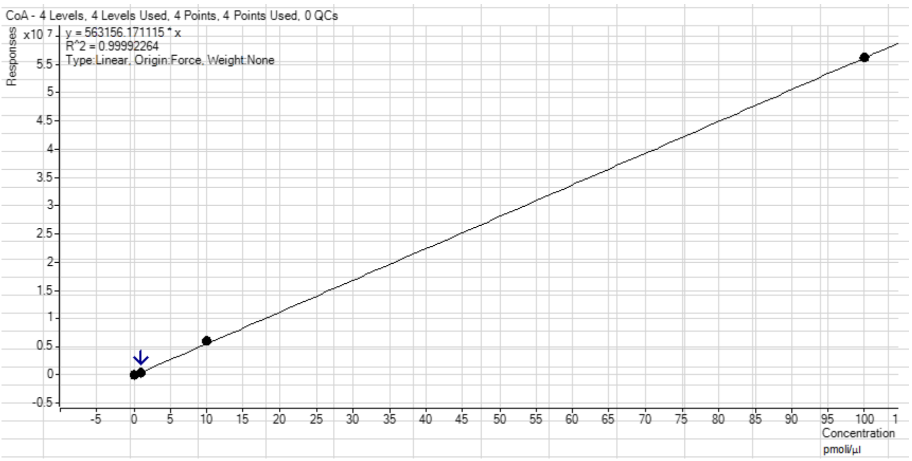

CoA - 4 Levels, 4 Levels Used, 4 Points, 4 Points Used, 0 QCs
x10 7
y = 563156.171115 * x
R^2 = 0.99992264
Type:Linear, Origin:Force, Weight:None
Responses
Concentration
pmol/µl

B

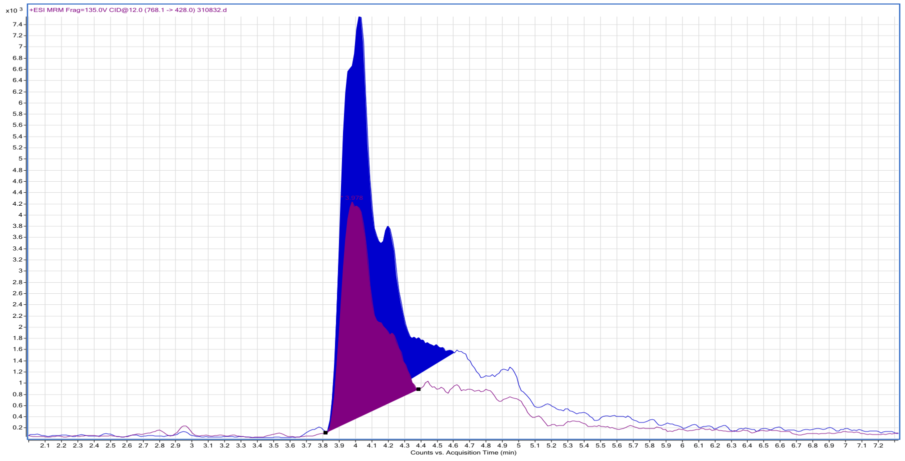

x10 3
+ESI MRM Frag=135.0V CID@12.0 (768.1 -> 428.0) 310832.d
Counts vs. Acquisition Time (min)

Figure S6

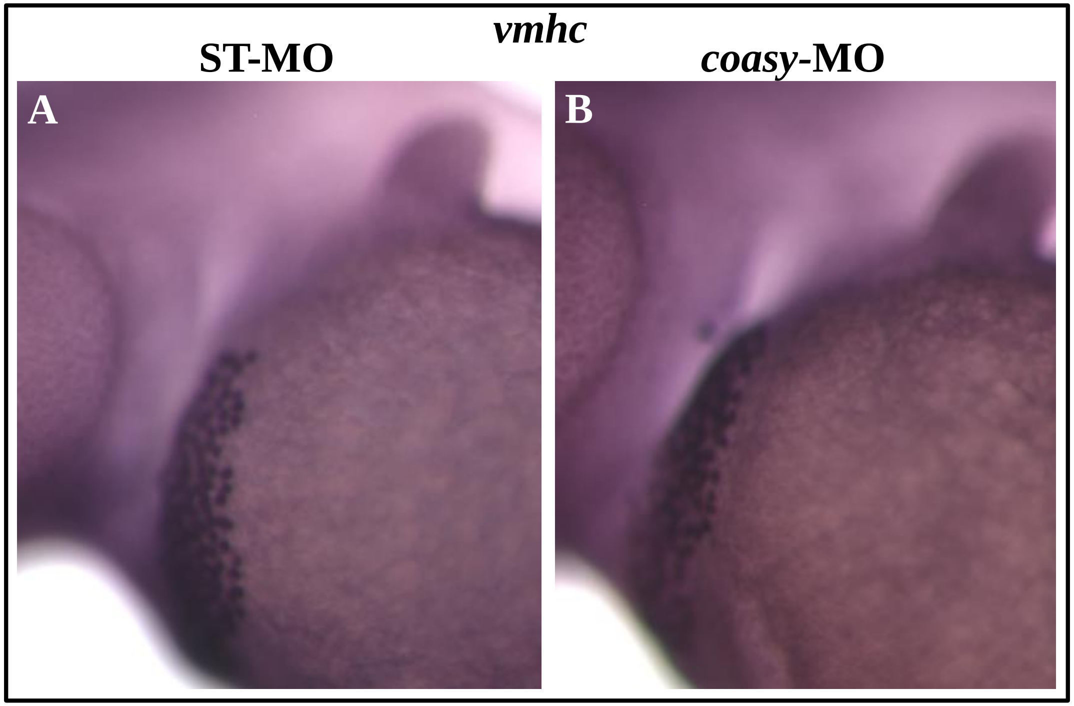

Figure S7

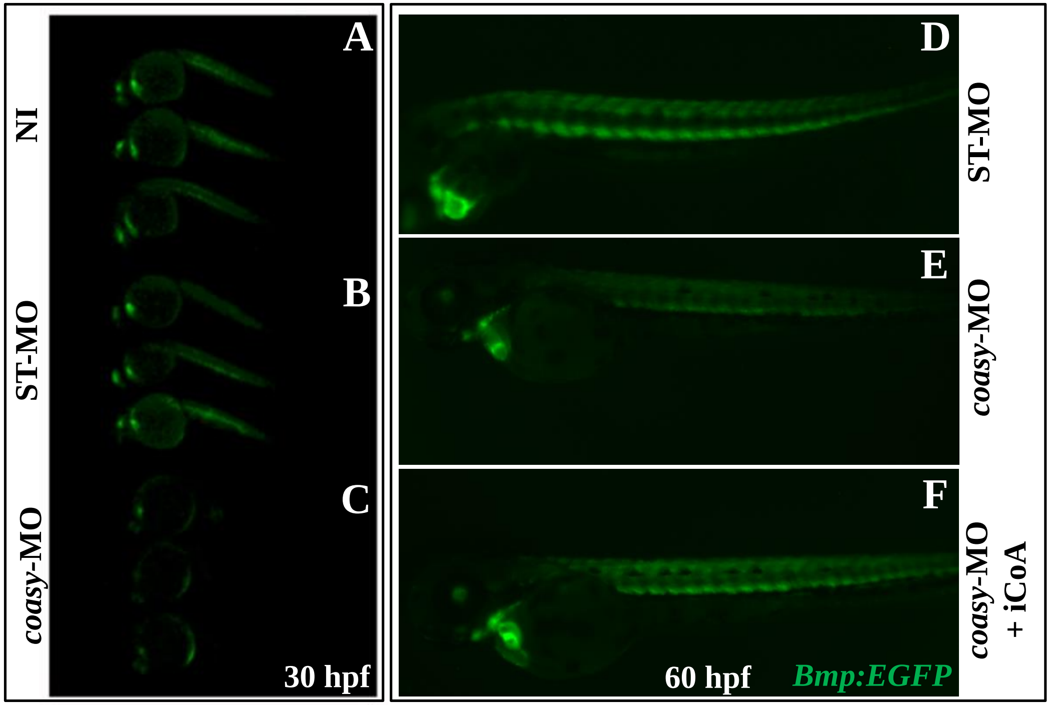
A
B
C
NI
ST-MO
coasy-MO
30 hpf
D
E
F
ST-MO
coasy-MO
coasy-MO + iCoA
60 hpf
Bmp:EGFP

Figure S8

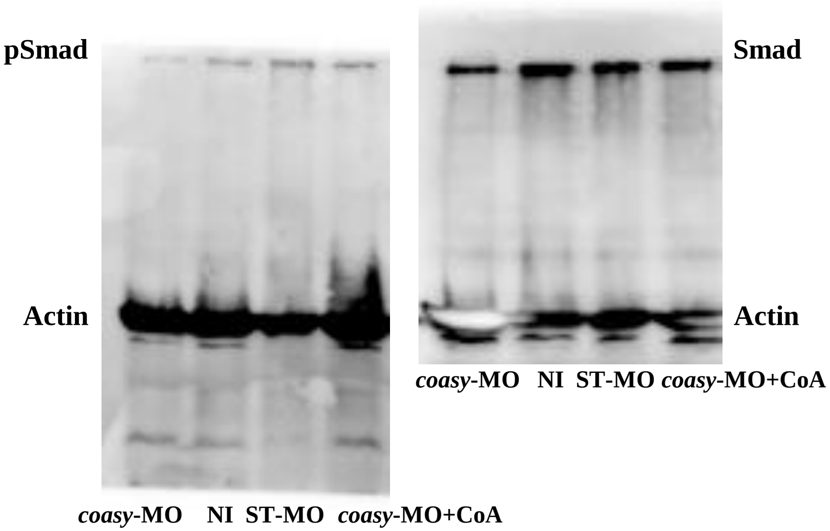

pSmad
Actin
coasy-MO NI ST-MO coasy-MO+CoA
Smad
Actin
coasy-MO NI ST-MO coasy-MO+CoA

Figure S9

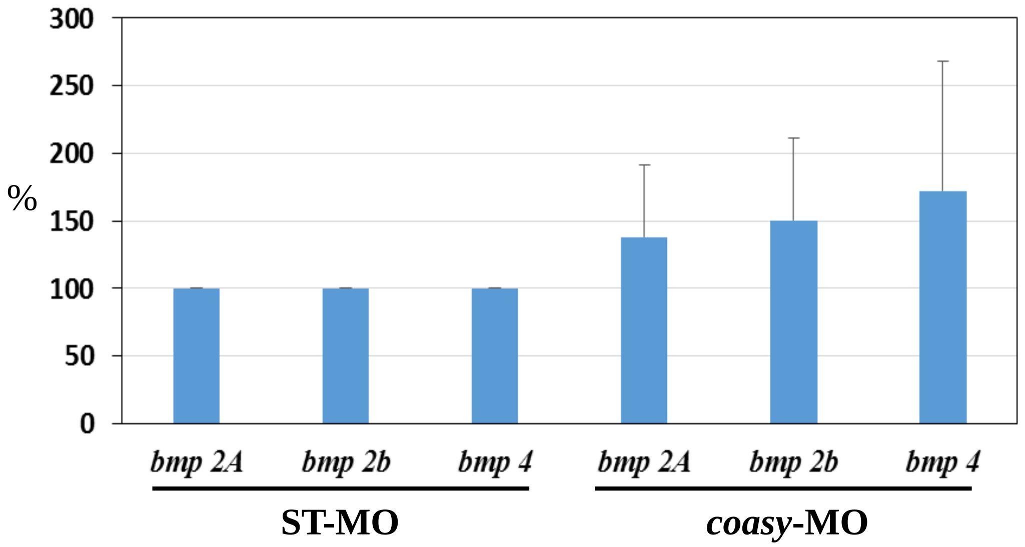

%
300
250
200
150
100
50
0
bmp 2A
bmp 2b
bmp 4
bmp 2A
bmp 2b
bmp 4
ST-MO
coasy-MO

Figure S10

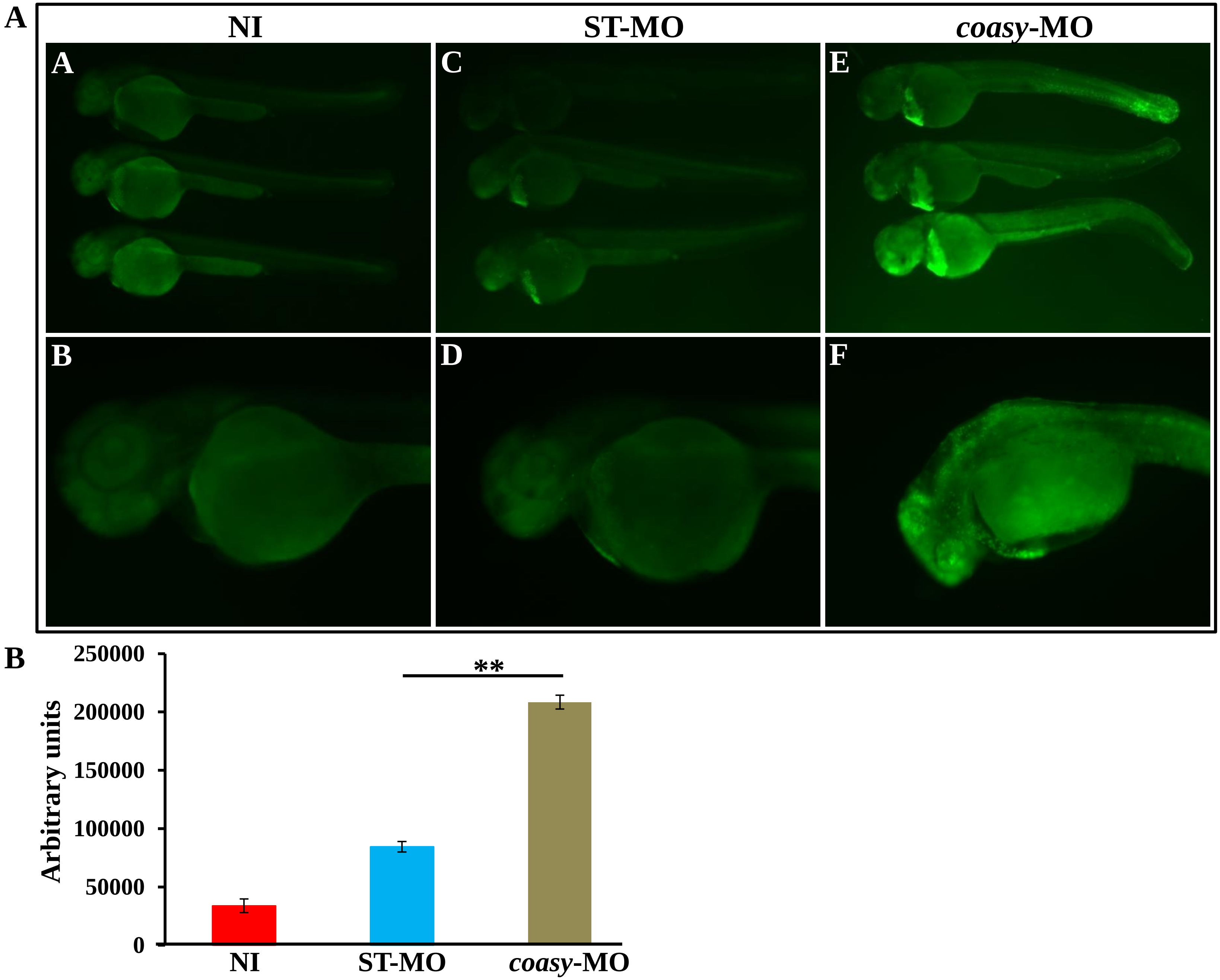
A
NI
ST-MO
coasy-MO
A
C
E
B
D
F
B
250000
200000
150000
100000
50000
0
Arbitrary units
**
NI
ST-MO
coasy-MO

Table S1

	48 hpf			60 hpf		
	H-T	Y-T	E.D.	H-T	Y-T	E.D.
**WT**	2.90 ± 0.17	1.82 ± 0.15	8,2 ± 0.10	3.80 ± 0.17	1.88 ± 0.14	1.0 ± 0.1
***coasy*-MO**	1.76 ± 0.19	0.82 ± 0.11	0.39 ± 0.10	2.25 ± 0.18	1.18 ± 0.2	5,5 ± 0.08

Table S1 Morphometric measures.

Head-Trunk (H-T), Yolk-Trunk (Y-T) and Eye Diameter (E:D) size were measured in 10wild-type embryos and 10 *coasy* morphants (1.2 pmol/embryo) at 48 and 60 hpf. Mean plus standard deviation is shown. All measures are in mm and P<0,0001 for all comparisons.

Table S2

Oligo		Sequence
coasy-MO	*coasy* morpholino	ACCACCTGAACATAGACATACAGCA
ST-MO	standard morpholino	CCTCTTACCTCAGTTACAATTTATA
QP1	*coasy* real time RT-PCR	CATTGGCTCTTCAGTCTCCTC
QP2	*coasy* real time RT-PCR	AGGTTTGGGTCGCAACTATC
A1	*actin* real time RT-PCR	CGAGCAGGAGATGGGAACC
A2	*actin* real time RT-PCR	CAACGGAAACGCTCATTGC
P3	cloning of *coasy* probe	TCCGGAATTCACCTCCACTGTCTGACCCCTT
P4	cloning of *coasy* probe	TCCGCTCGAGTCAGAGTGAAGATCTTGTCTCT
P5	*coasy* RT-PCR (exon 1-3)	GTCCTCACGTCCCCATTACA
P6	*coasy* RT-PCR (exon 1-3)	GTTTTGCACACGCCTCTTGT
P7	*bmpR1AA* RT-PCR	TGTCTTCTTCTCACACTCTGCTC
P8	*bmpR1AA* RT-PCR	TGTTCTTGGCATCATCGGGG
P9	*bmpR1AB* RT-PCR	GTGTTAAAAGCATGCGGCCA
P10	*bmpR1AB* RT-PCR	TGCCCCCATAGGTCTGGTTA
P11	*bmpR1BA* RT-PCR	GAGACGCTGAACAGGAACCA
P12	*bmpR1BA* RT-PCR	TTGGGGAAGGAGGGTCGTAT
P13	*bmpR1BB* RT-PCR	GCGCTACAGTATGGGTCTGG
P14	*bmpR1BB* RT-PCR	CTCGTGTCTCATCAGGACGG
P15	*bmpR2A* RT-PCR	CATCTTCTTGCCTGGTTGGACT
P16	*bmpR2A* RT-PCR	AAAGACAAGTCCTTCCCCACG
P17	*bmpR2B* RT-PCR	GTACTACCCTCATGGCTCGC
P18	*bmpR2B* RT-PCR	GTGCGAAATGGCAGGTTTGT
P19	*bmp-2A* RT-PCR	CAGGTTAGCAGACCCAGAGC
P20	*bmp-2A* RT-PCR	AACTCCTCGTCTGGGATGGA
P21	*bmp-2B* RT-PCR	ATGTAGAAAGGGCAGCCAGC
P22	*bmp-2B* RT-PCR	CTCCGAGAACTTGGTCCCTG
P23	*bmp-4* RT-PCR	CAGCACGTCAGCTTCGACTA
P24	*bmp-4* RT-PCR	TGGCGCCTTTAACACCTCAT

Table S2. Sequences of oligo-morpholinos and primers described in the manuscript.

SUPPLEMENTARY FIGURE LEGENDS.

Figure S1. ***In silico* analysis of *coasy* zebrafish gene**

A) Multiple sequence alignment of human (Hsa) and zebrafish (Dre) COASY polypeptides obtained using the MUSCLE software. In the ClustalW output format asterisks indicate positions which have a conserved residue, colons indicate conservation between groups of strongly similar properties, while periods indicates conservation between groups of weakly similar properties. Residues are colored according to their physicochemical properties.

B) Composite cluster representation of conserved synteny around the *COASY* locus between *Homo sapiens* chromosome 17 (Hsa17) and *Danio rerio* chromosome 24, generated using the Synteny Database (100-gene sliding window). Genes are drawn as squares with their order, but not their physical location preserved. Colored squares are members of the cluster while grey squares represent genes in the interval but that do not have orthologs (or paralogs) in the other segments. Lines connecting squares between the two clusters represent orthologous or paralogous gene pairs. Eight gene pairs can be identified in this orthologous pairwise analysis performed based on *Danio rerio* Zv9 and *Homo sapiens* GRCH 37 genome assemblies.

Figure S2. Expression pattern of *coasy* transcript during zebrafish development

Spatiotemporal analysis of *coasy* expression by WISH using a *coasy*-specific probe. WISH was performed from 0.2 hpf (1-2 cells) to 36 hpf with a *coasy*-specific antisense probe. (A) Dorsal view, four cells stage; (B) lateral view, sphere stage; (C) dorsal view, germ ring. (D-G) 20, 24, 36 hpf lateral views; (G) 36 hpf lateral view, magnification of somites. For each stage, at least 30 embryos were analyzed in two independent experiments. mhb, midbrain-hindbrain boundary**;** h, hindbrain**;** hbv, hindbrain ventricle; tg, tegmentum; s, somites.

A digoxigenin-labelled, *coasy* sense probe was synthezised as described in Material and Methods and applied for in situ hybridization with embryos at different developmental stages (H-J). No specific labelling was evident at any stage. Rapresentative images from one experiment performed with at least 30 embryos and repeated twice.

Figure S3. Embryonal expression of *coasy* analyzed by Real Time RT-PCR.

Total RNA was extracted from different developmental stages (A) and adult tissues/organs (B) and analyzed for *coasy* mRNA levels by real time RT-PCR. Results are expressed as relative quantification (RQ) and normalized to *actin beta 1* as endogenous reference gene. Data in graphs are from a representative experiment repeated twice with similar results.

Figure S4. ***coasy*-MO dose curve.**

To determine the efficiency of *coasy*-MO, we injected the morpholino at doses ranging from 0.5 to 2.5 pmol/embryo at one-two cell stage and compared them to embryos injected with the same amount of ST-MO. A) The graph shows the survival rate of embryos evaluated at 48 hpf. B) Representative agarose gel electrophoresis of RT-PCR products for *coasy* at 48 hpf from ST-MO (STD), *coasy*-MO-injected (MO), and not-injected embryos (NI) at different doses. Reduction of the upper band of 852 bp and appearance of the lower band of 281 bp is indicative of the altered splicing induced by *coasy*-MO.

Figure S5. Quantifcation of CoA concentration in *coasy* morphants by LC-MS/MS.

A) Representative calibration curve with 0.1, 1.0, 10, and 100 pmol/µl standard samples. B) The chromatogram represents the peak of the standard CoA (retention time 3.98 minutes) at a concentration of 1.0 pmol/µl.

The quantifier ion transition (768 → 261 in blue) and the qualifier ion transition (768 → 428 in violet) have been selected for the identification of CoA with high specificity.

Figure S6. *coasy* deficency does not affect heart development.

Stereomicroscope lateral views of WISH analysis with *vmhc* probe on ST-(A) and *coasy*-MO (B) injected-embryos (1.2 pmol/embryo) at 48 hpf. *vmhc* marks the hearth ventricle. The experiment was performed with 25 embryos and repeated twice.

Figure S7. Bmp activity at 30 hpf in the transgenic line Tg(*Bmp*:EGFP) and rescue by CoA injection

Representative images of Tg(*Bmp:EGFP*) embryos not-injected (A, B) and injected with 1.2 pmol/embryo of ST-MO (C, D) or *coasy*-MO (E, F) at 30 hpf. A net reduction of the fluorescence intensity is evident in *coasy*-morphants. Results are from one representative experiment with at least 38 embryos out of two independent replicates. CoA (0.5 pmol/embryo) was injected in the craniofacial area of Tg(*Bmp:EGFP*) morphants at 24 hpf (I) and Bmp activity compared to that of untreated embryos (G, H) at 60 hpf. One representative experiment out of two replicates.

Figure S8. Full size images of western blots for pSmad and Smad.

Full size images of western blottings shown in figure 8 D.

Figure S9. Real-Time RT-PCR for *bmp2a, bmp2b* and *bmp4*.

ST-MO- and *coasy*-MO-injected embryos were analyzed by qRT-PCR for *bmp2a*, *bmp2b* and *bmp4* expression levels. Data are shown as percentage of the value observed in ST-MO-injected embryos and represent mean and SD of three independent experiments.

Figure S10. Acridine Orange staining.

A) Representative images of embryos not-injected (A, B) and injected with 1.2 pmol/embryo of ST-MO (C, D) or *coasy*-MO (E, F) at 48 hpf obtained by acridine orange staining to assess the presence of dead cells. Morphants show a significant increase of the fluorescence staining, particularly in brain and tail. B) The quantification of the fluorescence intensity performed by a plate reader shows a significant ($P<0.001$), 2.5-fold increase in *coasy*-MO-injected embryos. Results are from a representative experiment with at least 50 embryos, repeated at least three times.
